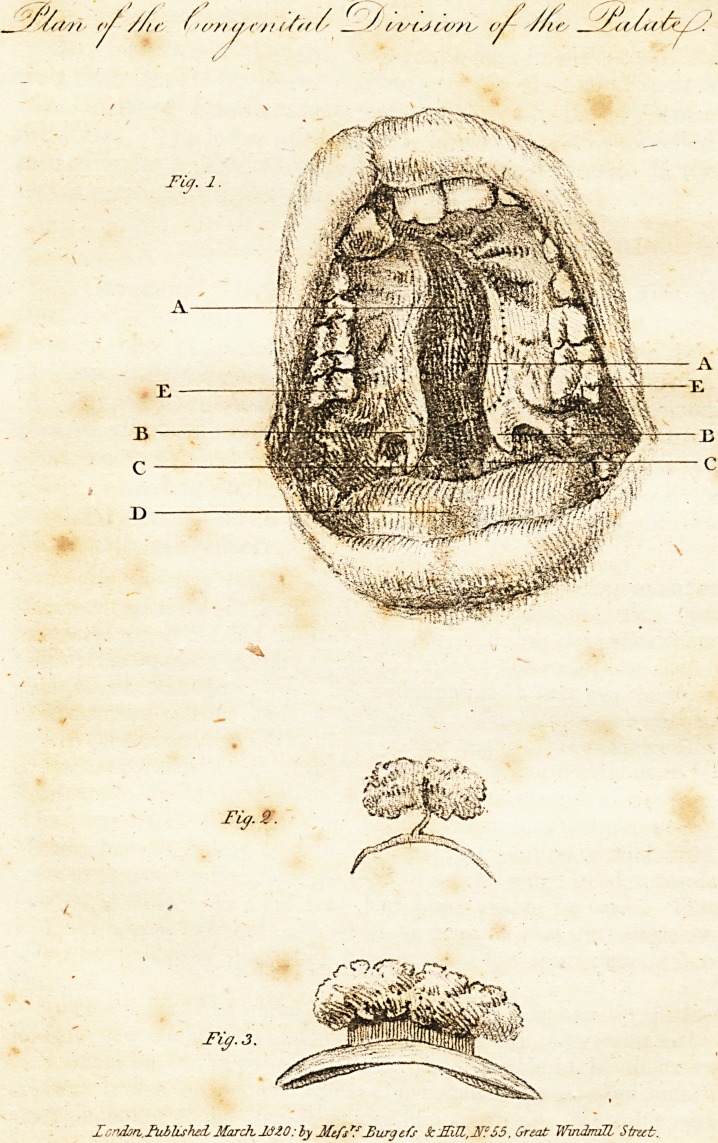# Supplemental Review of Practical Medicine, Digested and Arranged

**Published:** 1820-12-01

**Authors:** 


### Supplemental Review

OF

### PRACTICAL MEDICINE;

SELECTED AND ARRANGED, WITH COMMENTARIES.

*Paucis libris immorari et innutriri oportet, si velis aliquid trahere, quod in animo fideliter hæreat.* SENECA.

Duo vitia vitanda sunt in cognitionis et scientiæ studio. ***** Alterum est vitium, quod quidam nimis magnam operam conferunt in res obscuras atque difficiles, easdemque non necessarias. CICERO.

We now again resume our series of Supplimental Reviews, in which we shall occasionally glance at the original departments of our cotemporary journals in this country. There is no species of medical literature which more requires the healthful process of candid commentary than this, and none which has so generally escaped it, for what good reason we know not. It is not to be supposed, however, that we shall notice *all* the articles of this description which are monthly or quarterly presented for sale in the public mart. That would indeed be a Herculean task! We shall probably appear very fastidious to some of our brethren. To such we recommend a moment's reflection on the two quotations at the head of this article.

### § I. HEAD.

1. *Meatus Auditorius.** It cannot be concealed that we have made much less progress in the pathology and treatment of diseases of the ear, than of the eye or other organs of the body; nor is it likely that we shall ever be able to do much for those affections of the auditory apparatus which have their seats in the labyrinth, and deeper mansions of the seventh pair of nerves. We may yet, however, as Mr. Earle properly observes, be able to discover causes of deafness in

* On Affections of the Meatus Auditorius Externus. By Henry Earle, Esq. Assistant Surgeon to Bartholomew's Hospital, &c. *Med. Chir. Trans.* Vol. X.

the external ear and cavity of the tympanum, which have not hitherto been described, and which may partake of the nature of diseases affecting other parts of the body, and thus yield to appropriate remedies.

Early in 1816 Mr. Earle was consulted by a young officer, who stated that, from childhood, he had been subject to occasional attacks of inflammation in the external ear, accompanied by a copious thin discharge from the passage, and temporary deafness. An unusually severe attack of this kind, about ten months before Mr. Earle was consulted, had so far impaired his sense of hearing as obliged him to quit his regiment, for the purpose of obtaining relief. On examination, Mr. Earle found the meatus of either ear much narrowed in its calibre by the thickening of the surrounding parts, especially by the great increased density of the cuticle, which had a white appearance, and was moistened by a thin discharge resembling rennet whey depositing small portions of a curdy-looking substance. On clearing all away, and dilating the meatus, there did not appear any ceruminous secretion, but the same white thickened cuticle was seen to extend as far as the eye could reach. Although the sense of hearing was nearly gone, yet a watch applied to the teeth or forehead was distinctly audible, convincing our author that there was no defect in the auditory nerve. This naturally led Mr. Earle to believe that the deafness resulted from a thickened state of the cuticle reflected over the membrana tympani, or from the deposition of some morbid secretion there. When a probe was passed down, it did not produce the usual sensations in the patient, nor convey the feeling of a healthy state of parts to the surgeon's hand. Mr. Earle now determined on the removal of the whole cuticular lining of the meatus externus. To effect this he had recourse to the nitrate of silver, which he had found beneficial in causing exfoliation of thickened cuticle from the feet, producing what are called corns. A very strong solution was therefore thrown in with a silver syringe, which completely blackened the epidermis of the meatus. In a few days warm water was injected to loosen the exfoliations. They were detached in small portions at first, and subsequently in larger pieces, one of which, from its form, was evidently the reflected layer which covered the membrana tympani. After this, the injection of warm water caused a distressing sensation and loud sound. His hearing from this time was greatly improved, but still rather confused. The other ear was treated in the same way, and with similar success. In a few days the hearing was very nearly restored. From the separation of the cuticle, the treatment consisted in the application of ung. hyd. nit. ℥iv.

cerat. cetacei. ℥iij. ol. olivæ ℥j. misce. A little of this was introduced night and morning, on a camel-hair pencil, with the view of stimulating the ceruminous glands to a more healthy secretion. Blisters were also applied behind the ears, and kept open for some time. He rejoined his regiment, and has remained perfectly well.

From the silence of authors respecting cases of this kind, Mr. Earle has been induced to lay the above instance of successful treatment before the Medico-Chirurgical Society, accompanied by a train of very ingenious observations, explanatory of the pathology of the complaint. But, as we are only able to record the fact, we must refer to the volume itself for the reasonings.

Mr. Earle mentions two other cases in this paper, which we deem worthy of notice. The first was a gentlemen who applied to our author on account of a deafness of some continuance. On examination, the whole meatus was found choaked up with numerous scales, closely impacted together by means of a morbid secretion of cerumen. The patient had been subject, from youth, to attacks of lepra vulgaris, which latterly never entirely left him, and, at times, spread over nearly the whole surface of his body. Injections of warm fluids, and the subsequent introduction into the ear of an ointment composed of equal parts of ung. zinci, ung. hydrarg. nitrat. and cerat. cetacei, together with the internal use of decoct. sarsæ and alterative doses of the oxymurias hydrargyri, effected a complete cure.

The other case was that of a lady who never, from birth, had any proper ceruminous secretion. Our author found the meatus dry, and the substance deposited thereon exhibiting no character of cerumen. She had two brothers and a sister similarly affected. At times, after exposure to cold, or when in bad health, the secretion was more abundant and thinner. This irritated the passage, and the whole ear presented an erysipelatous redness, accompanied with considerable tumefaction and distressing deafness. The meatus being unnaturally narrowed, Mr. Earle directed a sponge tent to be used for the purpose of dilatation, after the local inflammation had been allayed, and while medicines were administering to improve the general health. By this plan the passage was considerably dilated, and her hearing much improved. She remained well for near a twelvemonth, when she again suffered a severe erysipelatous attack, accompanied by a copious ichorous discharge. After syringing the ears night and morning, and the inflammation had abated, the passage was anointed with the ung. hyd. nit. mitius. She improved rapidly, and remained in a comparatively better state afterwards.

These cases, as Mr. Earle justly observes, shew the important part which the ceruminous secretion acts in the auditory functions, and prove the necessity of paying great attention to this secretion, and to the state of the integuments lining the meatus externus. They also authorize us to hope that, by the application of suitable remedies, many distressing cases of deafness may be palliated, and some permanently relieved.

The thanks of the profession are due to Mr. Earle, who evinces a very laudable zeal in the advancement of surgical science, combined with a liberal and ingenuous tone of conduct and feeling, which is highly prepossessing in this young and rising surgeon.

2. *Meatus Auditorius.** The following extract we shall introduce in the words of the author :—

" There is an obstinate and troublesome disease of the ears, for which I have found the mercurial ointment an excellent remedy. I do not recollect to have met with a description of this complaint in any medical or surgical work. It is seated in the meatus auditorius, is attended with a severe and painful itching, a constant beating and ringing in the ears, and, at times, with a thin, acrid discharge, that excoriates and inflames the parts over which it flows. This discharge appears to proceed from a number of small ulcers which are spread over the surface of the meatus, and probably over the tympanum itself. The ulcers will sometimes of themselves heal up, and remain in that state for weeks, or even months, leaving behind no other indication of disease than an occasional itching; they will then gather, again break out, and continue to discharge for several months, to the great annoyance and distress of the patient. During the period of gathering, as it is familiarly termed by the patient, there is an extreme degree of tenderness for some distance round about the meatus externus, which forbids even the slightest touch.

" I do not know that this complaint has ever terminated in deafness, but in some cases, which have fallen under my observation, the hearing has been considerably impaired. I have known it alternate in one person with a painful heat and itching of the eyes. When in these organs, the ears were entirely free from complaint, and, on the contrary, when the latter were diseased, the former were perfectly well. Before using the ointment, I had been very much baffled in the treatment of this complaint. I had tried blistering behind the ear, and resorted to various stimulating and astringent injections, consisting of sulph. zinci, superacetas plumbi, sulph. cupri, &c. but all to no purpose. The ointment is the only remedy, which I have

* Dr. A. T. Dean, American Medical Recorder, July 1820.

found to have any control over this disease. By means of it, I have succeeded in curing several obstinate cases, which resisted every other mode of treatment. If it does not effectually eradicate the complaint, it will at least suspend, or cure it for a time." 306.

Dr. Dean thinks that this is a much less formidable affection than the "puriform discharge from the ears," which Mr. Curtis of London treats successfully with the argentum nitratum. The latter affection Dr. Dean has often succeeded in curing by repeated blistering, and a perseverance in the use of astringent injection.

3. *Congenital Division of the Palate.** Deformities of this kind, especially when they impede such important functions as those of speech and deglutition, are very serious misfortunes, and are generally very difficult to remedy or remove. Mr. Alcock's ingenuity was happily exerted in the following case, for the details and drawing of which his surgical brethren must feel much indebted to him. Mr. Alcock engraved the plate, as well as sketched the representation which we have obtained permission to republish from the April No. of the Medical Intelligencer.

"The patient, a youth under 12 years of age, had laboured under the inconveniences of the malformation from the time of birth. Not only were the uvula and the soft palate divided, but the palatal portion of the upper maxillary bone, and also of the ossa palati, were so deficient, that there was a free communication between the floor of the nostril and the roof of the mouth. The fissure extended to within $\frac{3}{8}$ths of an inch of the upper incisor teeth. The consequence of this malformation was, that the voice was so inarticulate as to be nearly unintelligible to those unaccustomed to his manner of speaking. The secretion from the nostrils drained down into the mouth, and his swallowing, unless performed with great caution, would often seem to threaten suffocation, the food or drink being forced upwards into the nostrils. As a child he had been unable to suck. This will not appear extraordinary when we consider that the soft palate acts like a valve in preventing what we swallow from passing into the nose.

"The patient had undergone the operation for hair-lip during infancy; but no attempt had been made to remedy the greater evil.

"From habit, the patient was but little sensible of his defect in speech; it was the inconveniences above stated that induced him to wish something to be done to relieve him of them. Even his near

* On Congenital Division of the Palate. By T. Alcock, Esq. Surgeon, Piccadilly, with a plate. (Med. Intelligencer, No. 6.) See plate in front of this number.

### Plan of the Congenital Division of the Palate.

Fig. 1.

Fig. 2.

Fig. 3.

London, Published March 1820: by Mess.rs Burgess & Hill, No. 55, Great Windmill Street.

relations had become so used to his nasal tones as not to be anxious on that account; and if any one imitated him he would exclaim, with great emphasis, and a nasel sound which defies expression by letters, " I oont eek oo!" (I don't speak so.)

" It was stated to his relations, that if the back part of the divided portions could be united, that an artificirl palate might then be made to fit the firm edges of the opening, and thereby wholly obviate the inconveniences under which he laboured. The latter part was that first attempted. I may here beg leave to notice, that the principle of supplying an artificial palate has been long admitted; but I am not aware of any work wherein the detail is so stated as to enable the apparatus to be constructed with sufficient accuracy; and unless it be accurate, the patient will soon discard it.

" The annexed sketch will convey some idea of the parts. The head was turned back, the mouth fully opened, and the tongue pressed down. See plate 2. fig. 1.

A. The cleft in the palate, opening into, or communicating with the nostrils, thereby making the mouth and nostril one common opening.

B.B. Part of the uvula, a portion being on each side of the cleft.

C. The amygdalæ, or tonsils.

D. The tongue pressed down.

E. The teeth, in outline.

The dotted lines represent the space covered by the artificial palate.

" The means I used on this occasion were similar to those employed by Dentists, in adjusting artificial teeth. I took a piece of plastic wax, and having given it somewhat the necessary form, I pressed it firmly upon the palate, so that the wax was pressed into the hollows, and received the impression of the eminences of the parts to which it was applied. This being carefully removed, was allowed to become hard, after which a cast was made from it in plaster of Paris. This cast fitting the wax model, as the model itself fitted the palate, gave an exact fac-simile of the form of the living parts; and to this was the artificial palate made perfectly to correspond, so that the adaptation was complete. To support this plate an upright slip was affixed, the upper part of which was covered with soft sponge. This readily passed through the fissure, and the sponge resting on the floor of the nostril, kept the apparatus steadily in its place. Care was taken that the sponge should not press on the edges, lest the aperture should be enlarged.

" Fig. 2. shews a section of the palate-plate to exhibit the manner of supporting the apparatus in situ. A duplicate should be provided to admit of cleanliness, as the sponge becomes saturated with the secretion from the nostrils in a few hours. Fig. 3. a side view. The dots represent holes to allow of the sponge being secured.

The voice was much improved, and his swallowing performed with greater facility. So satisfied were the boy and his parents with the relief which he experienced, that they declined the use of any further means to render it more complete. THO$^{s.}$ ALCOCK.

*3, Piccadilly, April* 1820."

### § II. CHEST.

4. *Disease of the Heart, &c.** There is probably not a more interesting or instructive case of complicated disease, of several important organs on record, than the following history presents; and therefore we shall give a very full analysis of it.

On the 25th September, 1818, M. Gogiran was called, in conjunction with M. Naudin, a distinguished surgeon of Toulouse, to Mademoiselle Fani, 30 years of age, the principal figurante of the Toulouse theatre. For six weeks previously, the following symptoms had gradually increased to their present state.

"*First visit:*—Countenance flushed, lips livid red, inability to lie down, breathing short and interrupted, pulsations of the heart very strong, and both visible and audible; pulsation of the large arteries, especially of the cœliac and its branches; the pulse at the wrist precipitous, irregular, and obscure; the abdomen painful, particularly in the right hypochondrium; urine lateritious; acute, and almost insupportable pain in the right shoulder; sleep short and interrupted; great emaciation; dull sound of the chest, on percussion, especially about the cardiac region." 343.

Who would not have considered the above symptoms as clearly indicative of hypertrophy, or active enlargement of the heart? This was the opinion of Gogiran, and also of Naudin, who had been a month previously in attendance; the supposition being strengthened by a consideration of the patient's mode of life, which subjected her to violent muscular exertions—a common cause of cardiac aneurisms.

The menses having been stopped for two months, a dozen of leeches to the pudendum, and the most rigid regimen enjoined.

29th. A slight diminution of the violent action of the heart, the other symptoms remaining stationary; but on the 2nd October, there was an increase of the cardiac action, with excessive pain in the shoulder, to which a tartar emetic plaster was applied.

*5th October.* Œdema of the feet and ancles, enlargement of the abdomen, increase of tenderness in the region of the liver, with much oppression. On the 9th, these symptoms still augmenting, pills, composed of the saffron of steel, calomel, and digitalis, were prescribed. 20th. There is manifest fluctuation of water in the abdomen, with great restlessness, oppression, paucity of urine, and derangement of the *animal* functions. Nitre, and tincture of digitalis, were now added to a diuretic beverage. 30th. Considerable amendment has taken place. The urine is more copious; the circulation

---

* Observation d'une maladie du cœur, suivie d'obstruction au foie, d'hydropisie ascite, et d'un état convulsif de l'estomac. Par M. M. Gogiran et St. André, docteurs en medicine, a Toulouse. [Societé de Medicine de Paris, séance du 17 Aout, 1817.]

more free; the action of the heart and arteries less strong and distinct; the pulse more developed and regular.

By the 4th of November the dropsical symptoms had nearly disappeared, and the rhythm of the heart had become nearly natural. But now, an enlargement of the liver (engorgement du foie) was evident. An irritating plaster was applied to the hepatic region, and the digitalis was increased.

Throughout the month of November there was a daily amendment, with excellent appetite and perfect digestion. All appearance of ascites and œdema had vanished; and the engorgement of the liver was somewhat reduced. In December, the action of the heart continued nearly natural—the sleep was sound, and the urinary secretion healthy. The patient could get up, and walk about her chamber: in short, throughout this month the health of the patient continued to improve daily.

Early in January, there were some symptoms of relapse; but by the end of the month, the patient was convalescent—went out—took exercise, and at length appeared in perfect health. Medical attendance was, of course, discontinued.

After two months of restored health, the symptoms began gradually to return, accompanied, this time, by distressing nausea and vomiting.

On the 20th April, M. Gogiran was called to the patient, and found the dyspnœa very great, with sleeplessness, violent action of the heart, particularly conspicuous in the epigastric region. The abdomen was now rapidly filling with water, ascites and hepatitis being very evidently declared. The digitalis, with aperients and narcotics, were again had recourse to. By the end of the month, the constant vomiting, the emaciation, and the cardiac disorder, had reduced the sufferer to a very low ebb. At this time, M. Gogiran being called away, she was delivered over to the care of M. Saint André, who noted the following symptoms.

"Considerable effusion in the abdomen, violent pulsation of the heart, as though it was aneurismal, right hypochondrium distended by the enlargement of the liver, the convulsive vomitings were so frequent, that they seemed to be only interrupted by alarming fits of syncope, inability to keep the least nourishment on the stomach, interrupted sleep, extreme dyspnœa, inability to lie down—emaciation to the last degree."

Opiates and antispasmodics were prescribed, with some other trifling remedies, and asses' milk. *6th April.* The milk seems to stay a little on her stomach; but this amendment was of short duration, and the vomiting, with all the other bad symptoms, were, if possible, aggravated, although she took eight grains of opium daily. Ice was now applied to the pit of the stomach. In four days, this remedy completely arrested the vomiting. She began to take nourishment, and the functions became more regular. The oppression from the ascites, was, however, very distressing. Afraid of renewing the gastric irritability, M. Saint André exhibited the tincture of digitalis by clyster, and by frictions on the skin of the lower extremities. In a few days, the urine was considerably increased, without any irritability of stomach being induced; the size of the abdomen diminished, and by the 6th May the water was entirely evacuated, the enlargement of the liver becoming proportionally more conspicuous. The dyspnœa, and the violent action of the heart subsided gradually, and the patient was soon able to take exercise. The frictions of the digitalis were continued, to which was added, mercurial friction over the region of the liver. By these means, the swelling of the right hypochondrium was reduced, and finally dissipated. In the month of June, the patient left off medicine, and undertook a journey to Paris, where, at the end of three months, she was seen in good health, with the exception of slight occasional irregularities of the circulation, at long intervals.

Messieurs Bouvier and Legumeau have made some interesting remarks on the above case, a few of which we shall notice here.

They consider, and we think justly, that the cardiac symptoms above enumerated, are to be attributed to *inflammation* of the heart, or pericardium, or of both at the same time. It is true, that *pain* in the region of the heart was not complained of; but every practitioner is aware, that inflammatory action often goes on to a great extent in an *internal* organ without the *pain* being sensible. The heart and pericardium have been found glued together by adhesive inflammation, in patients who have never complained of pain in that region during their lives. There is every reason to believe, that a certain degree of hydrothorax, or hydropericardium existed at certain stages of the foregoing malady. Indeed, on the 15th October, the chest, on percussion, gave strong evidence of watery effusion, although, on an examination some days previously, there were no such phenomena discoverable.

That HEPATITIS was complicated with the thoracic affection, in the above case, no man can doubt. The obstruction, or engorgement, however, does not appear to have involved any serious alteration of structure, but rather, a general distention of all the vessels of the organ, vascular, lymphatic, and excretory, which is remediable, and that, sometimes, very speedily, by the proper means. Whether the hepatic and cardiac affections, in this case, stood in the light of cause and effect to each other, and which was the original one, it would be desirable to ascertain. We know that both functional and organic diseases of the heart, will, sometimes, produce engorgement of the liver; and that congestions and enlargements of the liver will, on the other hand, embarrass the function, and sometimes impair the structure of the organ of circulation. In the case just related, we think the disorder of the liver preceded, and probably caused, that of the heart; and this is the opinion of the reporters on the case. There can be little doubt, also, but that the palpitation and violent action of the heart, in the relapse particularly, were *nervous*, or symptomatic of the disorder in the liver, and not resulting from any serious organic lesion. They therefore lay open to us an important lesson and caution, relative to the difficulty of distinguishing functional, from structural derangements of the heart, and shew us how guarded we should be in our prognoses in this class of human afflictions.

In respect to the treatment, it was any thing but energetic, and this is acknowledged by the editors themselves. Neither the general nor local bleedings were carried to a proper extent. The disease of the liver was not treated with decision; and, upon the whole, we conceive, that the natural powers of the constitution, did more to restore this lady to health, than the prescriptions of her physicians. The effect of the ice, in stopping the extreme gastric irritability, is worthy of being borne in mind. We are also of opinion, that this lady will again relapse, sooner or later, if she does not embrace a very different kind of life from that in which her physicians found her.

5. *Cardi-hepatic Disease.** A weaver, 32 years of age, addicted to considerable intemperance, who had enjoyed good health till within three years past, complained to Dr. G. of great pain and oppression in the hypochondriac and epigastric regions, somewhat relieved by sitting in the horizontal position. No pain in either shoulder. An unusual and strong pulsation in the left hypochondrium, corresponding with that of the heart and arteries, which is 120 in the minute. Respiration rather hurried and oppressed, countenance pale, or rather somewhat livid, expression anxious, conjunctiva whiter than natural, tongue white and moist, bowels torpid. This was the 18*th October*, 1819. He was bled to 28 ounces, with temporary relief. A purgative of calomel and jalap, which brought off much lumpy fæces. On being carefully examined, while lying in bed, a tense and hard tumefaction was discovered in the epigastric and hypochondriac regions, which, on the slightest pressure, gave pain. The calomel and jalap were ordered to be repeated on several successive mornings, alternated with saline purgatives. He would not allow a blister or seton; was enjoined strict temperance.

Not much amendment being perceptible, a course of mercury, in the forms of pills and ointment, was prescribed on the 1st November, with a little more nourishing diet. From the 8th till the 20th, a profuse ptyalism took place, which exhausted the strength much, and was attended with œdematous swellings, dyspnœa, scanty urine and stools, insomnium, and other unpleasant symptoms.

Diuretics diminished the œdema; but the pulsation in the left hypochondrium continues great. The report on the 15th December, states, great fulness and tension in the hypochondria, and also in the line of the arch of the colon; breathing laborious, pulse irregular, with considerable convulsions. After rallying and falling back alternately, he died suddenly on the 7th January, 1820, after a too hearty meal of animal food.

* Dr. Gardiner, Ed. Journal, No. 65.

*Dissection.* Some effusion in the abdomen. Liver much enlarged and rather hard—the left lobe proportionally more enlarged than the right. The organ weighed 5¾lbs. Dutch, but the nature of the morbid growth not appreciable by the naked eye. Spleen enlarged and much indurated. No other disease in the abdominal viscera.

*Thorax.* Some effusion. Heart enlarged in all its parietes and cavities, resembling the heart of an ox, weighing 48 ounces.

This is a case of "active aneurism" of the heart, according to Corvisart, and "dilatation with hypertrophia," according to Laennec, complicated with enlargement of the liver. Dr. Gardiner does not appear to be acquainted with Laennec's work on the subject, and to it therefore we refer him for information, which will a little surprize him.

6. *Wounds of the Chest, and Empyema.** Penetrating wounds of the chest and abdomen, where are situated those great organs on which life immediately depends, have always inspired horror, and must always excite a lively interest, both as to their proximate and ulterior consequences. Few, or rather none, have ever had such a wide range of experience, in military surgery, as Baron Larrey. He has witnessed the carnage of almost every battle that has been fought during the revolutionary war, between the Pyramids of Egypt and the towers of Moscow. Any observations, therefore, from such an authority, are entitled to our undivided attention, and ought to be thankfully received by the profession at large. The paper under review is a very interesting one, and on a very important subject. We shall, consequently, exhibit a very minute analysis of its contents.

In the expedition to Egypt, anno 1803, Baron Larrey experienced the good effects of quickly closing wounds of the thorax, and carefully excluding atmospheric air from that cavity. Since that period he has had numerous opportunities of verifying the statements then made.

"The extravasation of fluids," says the Baron, "in the thoracic cavities, is much less dangerous than has been imagined. In fact, if it be not very considerable, it is soon isolated by a circumscribed cyst, the vessels (venous) of whose parietes either drink up ultimately the effused fluid, or suppuration ensues and gives vent to the matter, if not evacuated by an operation. The parietes of these cysts afterwards coalesce, and the patient is cured.

* Observation sur une plaie d'arme blanche à la poitrine, suivie de reflexions sur les effets de l'operation de l'empyème, que cette blessure a necessitée, et de plusieurs autres observations remarquables sur des plaies analogues; par M. le Baron Larrey. [Journal Complementaire des Sciences Medicales, Mai 1820.]

"But if the extravasation be great, Nature is unable to effect the process above described, and sinks in the effort—especially if the membranes surrounding the fluid lose their vital powers by the mechanical pressure of the blood on their surfaces, or perhaps from the deleterious effects of the blood itself on the extremities of the capillary vessels. The extravasated mass, remaining then stagnant, is augmented by the serous and purulent secretions of the pleura, now in a degree of inflammation. Under such circumstances, unless art interfere, and open a passage for these fluids, death will ensue from the impeded function of the lungs, and the patient will be carried off by suffocation. Here then the operation for empyema becomes indispensible, in the sequel of wounds penetrating the thoracic cavities, as the sole mean of warding off a fatal issue." 195.

Having performed this operation with success in numerous instances, Baron Larrey considers himself authorized to say, that it is by no means dangerous, if properly performed. The great object, or rather difficulty, is to know, 1mo. *when* the extravasation is such as cannot be absorbed; and, 2ndo. *what* is the proper period for performing the operation? Without pretending to solve these questions completely, our author offers the following sketch of his reflections on the subject.

"1mo. In what case is the operation for empyema indispensible, where wounds have penetrated the chest? It is (as before observed) where the sanguineous extravasation is too great for absorption, which circumstance must be ascertained by the lapse of time since the infliction of the wound, (generally about nine days) and by the progress and intensity of the symptoms usually indicative of such effusion.

"Thus, in a penetrating wound of the chest, if any of the large vessels are opened, and one side of the thorax filled with effused blood, the operation becomes indispensible, particularly if the wound be situated at an elevated point of the chest. The symptoms denoting this extravasation will be oppression, difficulty of breathing, immobility of the ribs below the wound. To the pain which the patient feels when the surgeon presses his finger between two ribs, will be added a sense of undulation over the finger of the latter. The patient will not be able to lie down on the side opposite the extravasation, the motion of which will often be sensibly felt by the patient himself, or heard by the bye-stander.* Percussion too, though often equivocal, will sometimes assist us. Finally, this effusion is characterised by an œdematous ecchymosis behind the hypochondrium of the side affected. This our author considers as a pathognomonic

---

* "This phenomenon was very distinct in the case of a grenadier affected with hydrothorax in the right side of the chest. When he lay down on his back he could undulate the fluid in such a manner that it was distinctly audible at some distance. A seton, and several applications of moxa induced an entire resorption of the effused fluid. The parietes of the cavity gradually coalesced, and the soldier, who was presented to the Society of Medicine, was completely cured." 196.

sign, never having known it absent:—all the others are, more or less, equivocal,

"2ndo. At what period should the operation be performed, supposing it to be indispensible?

"It would be unsafe to perform the operation too early, because it might lead to fresh hæmorrhage, and disturb Nature in her attempt at resorption. We should be pretty certain that the divided vessels are consolidated, and that the effects of the fluid's *pressure* on the internal organs and linings of the thorax are developed. These effects do not generally manifest themselves, in such a manner as to be distinguished from the *primary* effects of the accident, till between the fifth and ninth day. Probably the operation will seldom be necessary before the seventh, or successful after the eleventh or fifteenth day." 197.

The following case is peculiarly interesting, especially as it discloses to our view what is not very likely to be often seen, death having unexpectedly taken place at the very moment when the man might be considered as almost cured.

*Case* I. Louis D***, 22 years of age, a grenadier in the second regiment of royal guard cavalry, of athletic constitution, irritable and headstrong, received, in a duel, on the 7th September 1818, at five o'clock in the morning, a wound by a cavalry sword, which went right through the upper and left side of the chest. His adversary experienced much difficulty in withdrawing his weapon. A terrible hæmorrhage followed; yet Louis fell not, but actually walked unassisted to a house, near the place of combat, whence, his wounds being bound up, he was immediately conveyed to the military hospital of Gros-Caillou. Here Baron Larrey found the patient, with scarce a symptom of life, from the vast loss of blood. Having stripped and chafed him with warm diluted vinegar, the dressings were removed, but with great caution, so as not to permit the air to pass into the chest. The instrument had entered between the first and second rib on the left side, and came out between the superior posterior angle of the scapula and the third dorsal vertebra. The edges of both wounds, and also the neighbouring parts were tumefied and emphysematous, in consequence of the external mouths of both wounds not being parallel with the wounds of their corresponding intercostal muscles, and also from the awkward application of the bandages. Nevertheless, whenever pressure was taken off the wounds, the blood flowed in jets from them, followed by symptoms of suffocation and approaching death. Baron Larrey concluded that the weapon had, in its passage across the chest, divided the internal mammary artery in front, the intercostal artery behind, piercing the superior lobe of the lung, and grazing over the arch of the aorta.

Our author's first object was to restore the parallelism of the wounds, by cutting some bands (brides) or shreds that kept their external and internal orifices in a contrary position. The lips of the wounds were then carefully brought together, and retained so by adhesive straps and bandages. Chicken broth with nitrous emulsion only for nourishment.

Cupping glasses with scarifications round the wounds soon removed the emphysematous swellings, and the hand of death seemed arrested, at least for the present. Frictions with warm camphorated and stimulating oils were applied over the body—the patient expressed himself as better—the breathing became less laborious—the pulse and heat more developed—the lips resumed their natural colour—and in a few hours every symptom announced a cessasion of the internal hæmorrhage.

In the same evening a febrile irritation ushered in a degree of reaction. The patient was therefore immediately bled, and cupping glasses were applied to all the left side of the chest. The dressings were not moved, and the night was passed calmly.

Next morning the pulse was found sharp and quick, with morbid heat of skin, redness of the cheeks, acute pains in the region of the wound, spitting of black blood, in considerable quantities. Venesection was repeated, diluent anodyne emulsions for drink, and laxative clysters exhibited.

"The third day, and during the night, the posterior wound opened, and an effusion of black blood took place. This was renewed on removing the dressings. This discharge of blood, far from soothing the patient, exasperated the nervous symptoms, and the unfortunate soldier all but perished among our hands, this time."

Both wounds were again secured, as well as possible—the patient was placed gently on a fresh bed—and the frictions again renewed over the surface, to elicit the circulation of the blood. From this till the 7th day the patient remained calm and comfortable, some blood escaping, from time to time, but without any air gaining access to the thoracic cavity.

On the night of the 7th day, the patient began to exhibit an extreme restlessness, without being able to enjoy a moment's repose, On the 8th day the pulse was quick and contracted; yet there was not much oppression; the respiration was not laborious; and the patient did not complain of any pain. Nevertheless an attentive examination of the chest, and of the patient's state altogether convinced Baron Larrey that there was an effusion in the left cavity of the thorax, and he determined, in his own mind, to operate for empyema the next morning, if the symptoms continued the same.

The ninth day was that of clinical lecture. The patient was the subject of it, and several foreign surgeons were present, who, having examined the chest minutely, could not be convinced that there was any effusion, and did not approve of the operation. But the Baron was firm in his opinion, and the never failing pathognomonic symptom of ecchymosis with œdema in the posterior region of the hypochondrium, being evident, he determined to operate in the presence of them all.

Every thing being ready, a spot was chosen, in the most depending point of the affected cavity—"point le plus reculé et le plus déclive de la cavité malade," with the precaution not to have the wound of the integuments and that of the intercostal muscles in a parallel direction.—"Avec la precaution de ne point laisser de rapport entre la division des tegumens et celle des muscles intercostaux." Arrived at the second bed of the intercostal muscles, the Baron ordered a large vessel to be placed at the patient's side; he then divided the internal layer, and enlarged the wound with a button-pointed bistoury. In a few seconds the vessel, which held about five pints, was overflowing with a grumous liquor, resembling, in colour, the lees of wine.

A tent was introduced, to prevent the closing of the wound, and dressings were applied, having apertures for any discharge that might flow. The patient was placed in a fresh bed, and expressed himself as progressively relieved from the moment the effusion began to issue from the wound. At the close of the operation, the pulse became developed, and all the functions resumed their natural action. He fell into a refreshing sleep of some hours. Bouilli, a little claret, and some pectoral ptisan were allowed. Next morning, after the operation, the dressings were found moistened with the same kind of discharge which was drawn off the preceding day, and which continued to flow pretty copiously. About the ninth day the discharge appeared purulent, and without any smell, after which it gradually diminished in quantity. The wounds by the sword healed slowly, especially the anterior one.

During the first month the patient manifested occasionally some febrile movements in the system, with gastric and nervous affections, for which were prescribed gentle vomits, cold infusions of bark, and light bitters. But a certain degree of emaciation followed these attacks, attended by some peculiar phenomena which are worth relating.

1mo. The beating of the heart, which had disappeared from its usual place when the effusion occurred, first reappeared at the left side of the sternum, and gradually moved its place till it resumed its original place of manifestation. From thence it gradually receded backwards and inwards till it became scarcely perceptible, at the epoch when the patient might be considered as almost cured.

2ndo. The pulsations of the radials in the two arms presented a striking difference. Those, for instance, of the left arm were distinct, regular, and uniform; those of the right arm were confused by a kind of second, or intervening demi-pulsation, presenting a character of undulation and retrograde locomotion.

3tio. The veins of the left arm were not apparent, while those of the right continued turgid and prominent during the first 25 or 30 days after the wound was inflicted. Ultimately, however, the pulse became synchronous and uniform in both arms, except that it remained a little weaker in the left than in the right side. These are curious phenomena for the physiologist to solve. The solution which Baron Larrey offers, does not appear very satisfactory, or even intelligible to us. As it is only a short passage, however, we shall give it in his own words.

"Il serait bien difficile de résoudre un tel problème; cependant ne pourrait-on pas croire que, par l'obliteration presque totale du poumon gauche, le sang destiné à son artère principale, obligé de rétrograder vers le ventricule droit, formait un obstacle au passage de celui contenu dans l'oreillette qui lui est contiguë; et de proche en proche, celui des veines qui s'y rendent, et surtout de celles qui parcourent le membre abdominal droit et le restant de la moieté droite du corps, pouvait rétrograder par une sorte de regorgement, jusqu'aux capillaires artériels, et déterminer ainsi dans les artères du bras un léger mouvement antipéristaltique, lequel pouvait agir, alternativement, avec celui (péristaltique) déterminé par la contraction directe et excentrique de ces mêmes artères?" *Journal Complimentaire, Mai,* 1820. *P.* 203.

Having passed the 40th day, the patient felt himself get better and better. The pus which came out of the wound was healthy and in small quantity—and the sword wounds were now perfectly cicatrized. The side of the chest affected assumed a size, position, and proportion below that of the healthy one. The left nipple descended an inch beneath the level of the right, the shoulder was depressed in a corresponding ratio, and the intercostal spaces on the left side were considerably reduced. The right side of the chest was enlarged in the same proportion.

Arrived at the 100th day from the operation, the patient ate light food, and walked several hours daily in the wards of the hospital, without any assistance. In short, at the end of four months there was every prospect of a speedy and perfect re-establishment of health. But this soldier, who was of a proud, fiery, and irascible disposition, became tired of the ennui of an hospital and its abstemious regime, delivered himself up to every excess which came within his power, especially the abuse of spirituous liquors, which he clandestinely procured, and which produced in him so much irritation and excitement that a violent inflammation of the heart and its envelopes was kindled up, and death ensued in 48 hours from the commencement of the disease. As the patient presented some symptoms during this carditis which are seldom enumereted by authors, we here transcribe them.

"The patient experienced a compressive and constant pain in the præcordial region, with a sensation of burning heat, and inextinguishable thirst. He threw himself, every minute, into violent paroxisms of anger—manifested a constant wish for death, and attempted suicide. He experienced severe cramps in the legs and feet; with icy chills running over his limbs. The pulse at the wrists was hard and contracted; but the action of the heart against the parietes of the chest was like the stroke of a ball of metal equal in size to the heart. The pupils were extremely sensible to the light. In fact this wretched man expired in a trsnsport of rage, and uttering all sorts of imprecations, on the 11th January, 1819, one hundred and twenty-five days after he had received the wound through the chest.

*Dissection.* The capacity of the left thoracic cavity was reduced one-third. The heart and pericardium occupied the greater part of the left side of the chest; the portion of lung which escaped compression, during the empyema, was hepatized, and connected by false membranes with the heart and pericardium—the two last being also intimately adherent. The diaphragm, on this side, was much arched, and helped to fill the space left by the fluid drawn off by operation. The ribs were thickened, and their interspaces shrunk in. A small quantity of matter was found in a little conical cyst, at the most depending part of the thorax, and communicating with the wound made in the operation, which continued fistulous. The two original wounds were completely healed; and on minutely tracing the course of the sword, from front to rear, the suppositions respecting the vessels and parts wounded were verified. The case, altogether, proves the perfect safety and success of the operation for empyema, even under the most unfavourable circumstances.

The following cases present examples of wounds penetrating the cavity of the chest, and accompanied by effusion, which being absorbed, a perfect recovery took place.

*Case* II. A cavalry soldier, 21 years of age, of strong constitution, and quiet character, was brought to the military hospital of Gros-Caillou, early in February, 1819, in consequence of a severe wound of the chest, which he had received in a duel a few hours previously. The cavalry sword had penetrated about three inches into the right thoracic cavity, entering between the fourth and fifth rib, a little behind the right nipple. Although he was speedily conveyed to the hospital, and immediately attended by Barron Larrey, yet the loss of blood had been great, and he was in an alarming state of prostration, blood of a vermillion colour, and frothy, still issuing from the wound in considerable quantities. The surrounding parts were tumefied and emphysematous. The patient was spitting up blood, his respiration laborious, the oppression extreme—in short, he appeared at the point of death. The Baron hastened to restore the parallelism of the wound, by dividing all bands of integuments that might obstruct the discharge of air or blood extravasated in the cellular membrane. Cupping glasses, exhausted of air, were then applied over the wound, after which the edges were carefully brought together, and secured by adhesive plaster and proper bandages.

The symptoms were now arrested, as by a charm, and the patient revived from the brink of death. The pulse, heat, sensibility, all gradually returned, and in a few hours every thing announced the cessation of the internal hæmorrhage. But the presence of an effused fluid in the cavity of the chest soon induced symptoms of irritation, inflammation, and compression, with difficulty of breathing, and immobility of the ribs of the right side. The patient lay on his back, or on the side affected; and, finally, a slight ecchymosis manifested itself, on the 3d day, in the posterior part of the right hypochondrium.

The patient was now bled, and enjoined cold mucilaginous drinks, while cupping glasses with scarifications were applied all over the right side of the chest. These means arrested the progress of the inflammation, but the symptoms of effusion continued. The wound was kept carefully closed, and the means abovementioned were persisted in. On the fifth day, there was little hope of resorption of the fluid effused, and it was determined to operate for empyema on the 7th, unless the symptoms ameliorated before that time.

In the night of the sixth day, however, after a violent attack of fever, a copious and fetid perspiration broke out, which proved critical; for, from that time, the symptoms of thoracic effusion diminished, and finally disappeared. The external ecchymosis also began to recede from the time of this perspiration, and gradually, though slowly, vanished—a proof to our author that the internal effusion was absorbed, *pari passu.* To aid this absorption, numerous flying blisters were applyed to the affected side, and five cylinders of moxa. All the functions became gradually re-established, and this young soldier was discharged from the hospital, in good health, at the expiration of three months from the time of his entrance.

Several other soldiers, belonging to the guards, who had received similar wounds, and in whom the evidences of thoracic effusion were equally strong as in the foregoing case, were treated in a similar manner, and recovered without the necessity for an operation.

*Case* III. A sergeant of the Swiss guards, named Placide C——, 21 years of age, of athletic constitution, was carried to the Military Hospital of Gros-Caillou, on the 23d of April, 1819, having received, in a duel, a sword-thrust in his chest. Baron Larrey was speedily on the spot, and found the soldier almost lifeless. The dressings were torn off, and two wounds appeared; one behind, between the inferior angle of the scapula and the spine, in the interval between the fifth and sixth rib;—the other wound, of a much smaller size, was near the left nipple. The sword, which was more than an inch in breadth, in the middle, had entered at the posterior wound, and emerged from the anterior. Our author concluded, from the direction which the sword had taken, that it must have pierced the pericardium near its adhesion to the diaphragm, though without wounding the heart itself.

"In paroxysms of rage," says Baron Larrey, "and in all violent passions of the mind, the function of absorption appears to be suspended, and consequently an accumulation of fluids obtains at these times in the serous cavities. Thus it is possible and probable that the liquor pericardii had accumulated in the pericardium during the rage of this combat. Acute hydrocele affords proof of the above reasonings, and the phenomena which we shall state farther on, confirms the truth of it." 209.

The space between the two wounds was found gorged and emphysematous. The introduction of a sound into the anterior wound, although with the greatest degree of gentleness, caused a sensation of anguish and suffering, yet without distinct pain.* Both wounds

* This was most probably from coming in contact with the heart. *Rev.*

were cleared of all cross bands, cupping glasses were applied over and around them; and then they were closed and carefully secured from the external air. The hæmorrhage appeared at length to cease —the patient began gradually to revive, and the vital functions finally became developed.

Evening.—The symptoms of great turgescence and irritation, with spitting of blood, having been manifested, the patient was bled, and two laxative clysters were thrown up.

*April 24th.* This morning the patient complains of great pain in the course of the anterior wound, with oppression, restlessness, flushings of the face, quick pulse, ardent thirst, and nervousness. All the dressings, except the adhesive straps, were removed from the anterior wound, and a poultice was applied, while blood was taken by cupping from the hypochondrium and lower belly. A large bleeding from the arm was also employed, with plenty of diluents, icy cold. The patient became somewhat more calm; but he was in imminent danger, and no one could entertain hopes of saving his life. The surgeon-major of the regiment, Dr. Hedellofer, announced to the colonel of the regiment, that the case was lost, as the heart or pericardium had been wounded.

25th. On the night of the 25th, a severe paroxysm of fever occurred, during which the surgeon in attendance bled him largely, and soon after a profuse perspiration broke out, and was succeeded by a calm.

28th. The dressings were completely removed. From the posterior wound the discharge was very trifling; but from the anterior one, a large quantity of an albuminous fluid issued spontaneously, followed by a shrinking of that side of the chest,. and an increase of freedom in the respiratory function, and a return of the cardiac pulsations against the ribs, the disappearance of which, for some days, Baron Larrey naturally attributes to the presence of a fluid accumulated between the heart and the anterior parietes of the chest. All the other symptoms of thoracic effusion now gradually disappeared; but this time the patient complained of a violent pain stretching from the posterior wound to the top of the corresponding shoulder, owing probably to the lesion of some branches of the spinal nerves. This pain was removed by cupping and warm anodyne embrocations.

From this period till the 4th May the patient continued to improve; but now again some symptoms arose indicative of a fresh collection in the præcordial region. The cautious introduction of the sound through the anterior wound, gave vent to half a palletful of sero-purulent fluid. The cardiac pulsations which had again disappeared, were now, once more, renewed, and the freedom of the respiratory function re-established. The discharge from the anterior wound became more purulent, and of a better quality, as well as diminished quantity; and, to the surprize of all the medical attendants, this young soldier completely recovered in 41 days from the infliction of the wound. At present (20th March, 1820) this man enjoys good health. The left arm is smaller and weaker than the right—the wounded side of the chest is somewhat shrunk, and the left nipple about an inch lower than that of the other side. The cardiac pulsation is bounded to a spot immediately beneath the cicatrix of the anterior wound, as if the organ was attached by its pericardium to the bottom of the original wound.

*Case* IV. A voltigeur, in the 6th regiment of guards, about 23 years of age, was carried to the hospital of Gros-Caillou, on the evening of the 30th September 1819, severely wounded by a sword in the chest, during a duel, a few hours previously. The extreme exhaustion and danger, in which the wounded man seemed, induced the surgeon on duty to allow the rude dressings to remain on the wound. Perceiving no external hæmorrhage, he endeavoured to restore the vital spark which appeared nearly extinct.

Next morning, at Baron Larrey's visit, the patient was examined, and a wound, an inch in diameter, was found under the right nipple, penetrating in a direction inwards and backwards, giving vent to vermillion-coloured and frothy blood, which was also thrown up by the mouth. The face was pale, the breathing short, oppressed, and laborious; the lips were white, the pulse gone, the extremities cold, the speech scarcely audible—in short, the patient appeared in articulo mortis. The sabre had penetrated into the chest to about the depth of two inches, dividing the cartilage of the third rib, near its junction with the osseous portion of the same. The two anterior branches of the intercostal artery were wounded, the superior lobe of the lung entered, and doubtless the phrenic nerve pricked, in its passage on the surface of the mediastinum. This was deemed to be the case from the depth and direction of the wound, and the nervous symptom which occurred, as soon as the vital forces returned, to wit, the pain, convulsive laughter, and subsequent severe neuralgia of the right arm.

Having cleared the wound of all cross-bands, and established its parellelism, a great quantity of crimson and frothy blood issued forth, and the patient appeared on the verge of death. They hastened therefore to close the wound, and keep its edges together by adhesive straps and bandages. In a few minutes, the pulse became perceptible, and all the vital functions were gradually re-established. Directions were left with the surgeon on duty to keep the patient on the most rigid regimen—to exhibit lavements—and to bleed whenever reaction set in. In the evening, blood-letting from the arm was copiously performed, followed by cupping glasses to the side, but without removing the dressings. The patient was kept in perfect quietude, with the head elevated.

The nervous phenomena, resulting from the supposed lesion of the phrenic nerve, continued obstinately, and the power of speech did not return for a long time. The pain in the arm and fingers was exceedingly acute, to which were soon added symptoms indicative of effusion in the chest, together with inflammation. Blood-letting was frequently repeated, and also the application of cupping-glasses. The wound was not opened till the fifth day, and then with great caution not to permit the entry of air into the thoracic cavity. By the ninth day the patient found himself much better. He could now speak a little, and the risus sardonicus began to diminish. Every thing announced that the work of absorption was going forward. Between the 11th and 13th day, however, traumatic fever rose, but was quelled by a blister to the side, and pretty large doses of calomel and opium. After this the patient went on favourably to convalescence. In two months, the wound was healed, the absorption of effused fluid far advanced, and that side of the chest evidently shrinking in. He soon recovered and was discharged from the hospital.

The minute analysis which we have given of Baron Larrey's paper renders all remarks from us unnecessary; as our surgical readers can form their own opinions, without any observations from us. We think, however, that the English surgeon will be gratified and instructed by the perusal of this interesting document from the pen of his able continental brother officer.

7. *Thoracic Affections.** In *acute* diseases of the thoracic organs it is often highly desirable to establish a counter-irritation on the skin, by a shorter process than blistering with the lytta—while, in *chronic* affections, a more permanent drain than that caused by vesicatories, is generally necessary. Our continental brethren are now employing the liquor ammoniæ, lowered more or less with oil, to effect both the abovementioned purposes, and this measure we particularly recommend to the notice of the profession in our own country. If a sudden impression is wished to be made on the surface, one part of oil to two of liquor ammoniæ will rapidly blister the skin, and produce a considerable discharge afterwards. If kept on a portion of surface, for instance, with a large cupping glass, for an hour or two, an eschar will be formed, and thrown off, leaving a complete caustic issue or drain, which may be kept open for any length of time, and give exit to a great and permanent discharge.

The more easy and practical way is, to cut a hole in a sheet of adhesive plaster, the size of the required issue, and after the plaster is placed on the part, let a few folds of linen wetted in the ammoniated oil, be applied over the whole, and retained there for two or three hours, after which, a poultice may be kept on till the eschar falls off. We shall

---

* *Reflexions sur l'emploi de l'ammoniaque, comme moyen propre à etablir des exutoires.* Par M. Vaidy, Journal Complimentaire, August, 1820.

here relate a case from M. Vaidy in elucidation of the effects of an issue of this kind.

"M. R. 50 years of age, presented to M. Vaidy, in the beginning of February, 1820, the following symptoms:—face pale, and yellowish, with some flush on the cheeks—tongue yellow in the middle and red at the sides—sense of heat in the throat—breath hot and fetid—breathing short and distressing when speaking—cough troublesome and in paroxysms, especially during the night—expectoration difficult, yet in considerable quantity, viscid, fetid, and apparently containing nuclei of purulent matter. Percussion of the chest caused pain, and elicited a dull sound below and behind the right nipple—no appetite—thirst moderate—some wandering pains in the abdomen—several bilious evacuations every day—pulse concentrated, and rather quick—want of sleep—great dejection of spirits. Had been about eight months ill. M. Vaidy learnt, that this patient had been subject to liver-affections—to a hæmorrhoidal discharge, and to a bilious diarrhœa. Our author prescribed a vegetable diet—pills containing extract of opium—and ordered 24 leeches to the right side of the chest. The bleeding afforded no relief—the right side of the thorax continued painful, and the cough was violent. The patient despaired of life, and the doctor of success. A seton or moxa was proposed, but neither was agreed to. The patient consented to a blister. M. Vaidy informed the patient that he would employ a liquid blister much more efficacious than the common one. He therefore applied, by means of a large cupping-glass, an ounce of the caustic liquor of ammoniæ, mixed with half an ounce of oil of almonds. The cupping-glass adhered for two hours, at the end of which, the skin was found cauterized throughout its whole substance, with an eschar two inches in diameter. When this eschar began to separate, the edges of the sore acquired an excessive degree of sensibility, while, at the same time, the symptoms, previously enumerated, became more and more alarming. The patient could not sleep at all, and was constantly in pain. When the slough came away, a large and deep wound was left, the surfaces of which afforded a very abundant suppuration. From this moment, the amendment was visible. The expectoration became less fetid, less copious, and less purulent. Both patient and physician began to entertain hopes. At this time, an eruption broke out over the body, accompanied with great itching. He had several liquid stools daily, notwithstanding the opium. At the end of two months, the sore appeared to cicatrize, but was kept open artificially. The health now began gradually to be re-established, and at the end of three months, he could sleep, eat, and exercise. The breath, however, continued a little short, and the patient has determined to keep the issue open till the end of the year."*

---

* We conceive that the above case offers an example of affection of the lungs, resembling, and often ending in phthisis, but produced by a disordered state of some of the abdominal organs. In the present

M. Vaidy conceives, and we agree with him, that the measure in question, is a most valuable resource when we have to deal with timid patients, who are afraid of setons or the moxa. He thinks too, that the ammoniated issue is preferable to either of these means.

*P. S.* Since writing the above, we have seen some communications from Dr. Kennedy in the last number of the Ed. Journal, on the use of nitrous acid, as a substitute for blisters, where a sudden counter-irritation is wanted. It has been used in India, by Mr. Killet and Mr Scott, for the treatment of the spasmodic cholera, and with advantage. They used two parts of acid to one of water, with which mixtnre, the surface is to be rubbed till the patient begins to complain of pain, when the acid is to be neutralized by washing the surface with a solution of salt of tartar. The cuticle can then be easily detached, leaving the cutis raw and denuded, to which, if necessary, the unguentum or emplastrum lyttæ may be applied to keep up the discharge.

8. *Hydrothorax.** A soldier, aged 32, had an attack of splenitis, which was removed by the depletory treatment, a few days after which, he complained of pain in the right side of the thorax, with cough and fever. These were relieved by the loss of 24 ounces of blood. About this time, the heart was observed to beat more to the right side than it ought, together with an unusually strong action in the aorta. He was discharged convalescent, but re-entered the hospital a few months afterwards, with great difficulty of breathing—pain and weight about the scrobiculus cordis, and along the whole left side of the thorax, frequent loose cough, pulse 108, small but regular, temperature natural, urine scanty and high-coloured, bowels slow, decubitus difficilis on the right side, rest broken by frightful dreams, sudden startings from sleep, feels the motion of a fluid in the left side of the thorax on turning suddenly, which sensation Dr. Hennen also perceived both by sound and touch. Diuretics were prescribed, with a purgative, which gave but temporary relief. Paracentesis thoracis was determined on, and performed, when instance, there is unequivocal evidence of considerable derangement in the biliary secretion, and also in the functions of the bowels. *Ed.*

* Dr. Hennen and Mr. Henderson.—*Ed. Journ.* 65.

four pints of a sero-purulent fluid were drawn off from an opening between the sixth and seventh ribs. The patient was immediately relieved, and passed a good night, which was followed by increase of appetite. The heart came more *in situ*, and its pulsations were obscurely felt in the left side. Diuretics were tried again, but left off; and by the 1st June, we find the patient with all his bad symptoms again, and evidence of a collection of fluid, the site of which was believed to be in the pericardium. On the 8th, the spleen was observed to be pushed down, by the weight of the fluid on the diaphram. On the 10th, he was apparently incapable of living many hours, and by way of Euthanasian solace, the operation was again performed, and eight pints of a fluid more purulent than before, were drawn off. The result of the operation was wonderful. He passed a good night, and next day, had an increase of appetite. A canula was left in the wound, and more or less fluid was evacuated between this and the 5th of July, when death released him from his sufferings.

*Dissection.* Left cavity of the thorax contained two pounds and upwards of matter, containing large flakes of coagulable lymph.

A pseudo-membrane, half a line in thickness, coated the whole cavity, and was thickly covered with purulent matter. The lung was much reduced in size, and would not float in water. The other lung was sound. Pericardium thinned, and adherent to the heart throughout its whole extent. The heart of its natural size, as also its vessels. Spleen of a natural structure, and perfectly *in situ*. Other viscera of the abdomen healthy.

*Remarks by Dr. Hennen and Mr. Henderson.*

Both gentlemen were surprized to find the heart healthy, as it was all along considered to be enlarged, and water in pericardio. "I am convinced, says Mr. Henderson, that the latter circumstance arose from our mistaking the small space that was unoccupied by the fluid in the chest, for the extended pericardium, and had we punctured it, as was at one time proposed, we should have done nothing more than we afterwards did, viz. evacuated the matter contained in the left side of the thorax."

Dr. Hennen makes many judicious and candid observations on this curious case. He had opportunities of seeing the patient at various periods, before and after paracentesis, and never doubted of the existence of water in the pericardium,

Dr. H. remarks that, at one time, when he examined the patient:—

"While he lay on his back the pulsations of the heart were strikingly visible on the *right* side, extending over a circle, the diameter of which, was four fingers' breadth, and its central point directly corresponding to the middle of the fourth rib. These pulsations at once suggested the heart beating in a bag of water, and the sensation conveyed to the hand, confirmed the opinion thus suggested to the eye, while the ear was forcibly struck with the sound of dashing water, occasioned by the patient's spontaneous motions. Although no one doubted, at the time, that *all* the fluid had been removed from the general cavity of the thorax, and that consequently this noise was produced entirely from the agitation of the fluid contents of the pericardium; yet dissection proved that a *part* of the water was prevented from running off by the interposition of the newly formed membrane; and the noise was no doubt greatly increased, by the admission of air through the puncture."

Dr. Hennen acknowledges, that he overlooked the possibility of adhesion of the pericardium to the heart being the cause of some of the symptoms, which it very often is.

Our author, after making many highly judicious and important observations on the case in question, relates another, which illustrates the difficulty of ascertaining the morbid conditions of internal organs.

A soldier, 25 years of age, was received into hospital on the 14th February, 1820, labouring under severe dyspnœa, with pain in the right side of the chest, and amazingly quick and irregular pulse. Bled till relief was experienced, which required the abstraction of three pounds. The good effects were soon apparent; for the temperature of the surface, before irregular, became now steady, and the pulse could be counted. The breathing, however, was still anxious, and he could not lie down. The heart palpitated violently and at each stroke, a fluctuation of fluid, within the thorax, was discernible. A large blister was applied over the seat of pain —feet immersed in warm water, and a purgative exhibited. When the bowels acted, the pain was relieved. No sleep that night. Next day the breathing was still laborious, but the pain had not returned. At night a violent spasmodic dyspnœa came on, and required venesection, which, however, failed to afford relief, and he lingered on three or four days, when he expired.

*Dissection.* Liver a good deal enlaged, and the gall-bladder filled with tar-like substance. The general cavity of the thorax was free from effused fluid, but 18 ounces of fluid were contained in the pericardium. The heart was small and pale. The lung on the right side was collapsed, of a dark sphacelated appearance, containing in its superior portion the sac of a large abscess, in which a small quantity of matter was still contained. The left lung was universally adherent to the pleura costalis, and its substance tuberculated throughout, or filled with vomicæ.

It is astonishing, as Dr. Hennen remarks, how this man could have existed, with such a mass of disease in the thorax, particularly in the lungs. It is also a case which exemplifies the remark of Baglivi.

"O quantum difficile est cognoscere morbos pulmonum!"

Dr. Hennen knows of only one case on record of hydrops-pericardii unaccompanied with effusion into the general cavity of the thorax, and that one is related by Sidren. It strikes us that we have seen some dissections of the disease.—Of effusion into this cavity, as one of the terminations of pericarditis, we have seen several, and cases innumerable are on record. But of genuine idiopathic hydro-pericardium unaccompanied by hydrothorax, three cases now lie before us, related by Dr. Romero, a Spanish physician, who opened the pericardium and drew off the water, twice with success. This operation he performed in eight cases, five of which were complicated with hydrothorax, and three were simply hydro-pericardium. As this gentleman's memoir is perhaps nearly unknown in this country, we shall present our readers with a very slight sketch of it here.

Dr. Romero, now a professor in the university of Huesca, in Arragon, formerly resided on the coast of Andalusia, where hydrothorax and hydro-pericardium are, as it were, endemic, from some peculiarity in the medical topography of the place. In Dr. Romero's opinion, the disease is there owing to a prevalence of hot and humid winds, with sudden atmospherical transitions. Ingurgitation and bad food are also blamed. Seeing that all the usual modes of treatment failed, Dr. Romero ventured on a most singular and dangerous plan, which was no less than opening into the pericardium itself.

Having first ascertained that the dropsical effusion does not depend on organic lesion of some neighbouring viscus, which would, of course, render the operation useless, Dr. Romero makes an incision between the fifth and sixth ribs, counting from above, close to the origin of the cartilaginous portion of the ribs, in people of middle stature, but between the fourth and fifth ribs, in people of small stature. This incision is carried through the pleura. Dr. R. then introduces his finger, and easily ascertains whether or not the pericardium contains a fluid. If it does, he makes an opening into it with a pair of small crooked scissors, and permits the fluid to escape into the general cavity of that side of the chest, whence it is discharged, by placing the patient in a proper position. If there be no water in the pericardium, but only in the bag of the pleura, the first incision serves to give it exit, and no opening is made, of course, into the pericardium. If he is deceived totally in his diagnosis, and finds no water in either the pleura or pericardium, then the wound is healed, and no bad consequence ensues.

Our author is well aware, that this operation gives but a temporary relief, as in paracentesis abdominis for ascites, and consequently he endeavours to remove the causes, and prevent the recurrence of the disease. When water is found in either of the positions abovementioned, the aperture in the pleura is kept open for four days, each day taking out the tent, and placing the patient in a convenient posture for favouring the escape of the fluid.

This operation has been performed, as we before stated, eight times, thrice for hydro-pericardium, and five times where hydrothorax was complicated therewith.

In conclusion, we may add, that Laennec, in his late valuable work on Mediate Auscultation, proposes that, in such cases, the sternum should be trepanned, as the easiest mode of evacuating water from the pericardium. There would be this danger, we apprehend, attending Laennec's operation, that air might possibly be let into the thorax, on both sides the mediastinum, in which event, death would be inevitable.

Since the above was written, we have looked into Leutaud, and there we find many cases of hydro-pericardium unaccompanied by effusion in the chest. In the first case, for example, there was a pint of water in the pericardium, and no mention is made of effusion elsewhere. In Obs. 612 (*lib.* 11) there were six pounds of serum in pericardio, one lung being gangrened, and the other suppurated. In Obs. 613, there were two pounds in pericardio, and the author distinctly states the absence of water in the chest. "Nulla aqua reperitur in cavitate pectoris." Obs. 415 and 416, from Morgagni, are to the same effect; in short, a great number of cases are here stated of hydro-pericardium, though a still greater number present complications of hydrothorax.

### § III. ABDOMEN.

9. *Fungoid Tumour in the Abdomen.** We shall introduce the following case in the words of our able author.

"William Norris complains of severe pain, extending from the spine of the right ilium downwards into the thigh, and sometimes towards the bladder. The pain, however, is not always equally violent, but subject to alternate paroxysms and remissions. There is a large tumour in the right hypogastrium, which, to the touch, appears solid. The body is much emaciated; the pulse quick, and exceedingly weak. Has never been troubled with vomiting, retraction of the testicle, nor the slightest symptom of hectic. The urine is high in colour, and very much loaded with mucus. The bowels require the frequent use of laxatives.

"Fifteen years ago he fell from a ladder upon his back, and has, ever since, been occasionally subject to slight attacks of pain in that part; from which, however, local applications always relieved him, until about twelve months ago, when the affection recurred with unusual severity. He then obtained admission into St. Thomas's hospital; and through the advice of Dr. Scott (one of the physicians to that Charity) underwent repeated cuppings and vesications, and used a variety of medicines internally, but (as he reports) with no other effect, than that of removing the seat of the complaint, to the spot it now occupies. Ordered anodyne fomentations, with balsam of copaiba, opiate injections and pills—and an aperient electuary occasionally.

"29th. Was seized with almost incessant vomiting of a green matter exactly resembling softened verdigris, to allay which the effervescing draught was ineffectually administered. The treatment was now wholly confined to opiates and anodyne fomentations, from the latter of which remedies he obtained a temporary mitigation of pain.

"30th. Sunk under the continual efforts of vomiting.

"*Dissection.* With the assistance of Mr. Young, a surgeon of this place, I proceeded to examine the body on the following day.

"It exhibited nothing unusual externally, except a swelling in the right hypogastrium, the skin of which was, in places, discoloured with extravasated blood.

"On laying open the abdomen, an enormous tumour presented itself, occupying nearly THE RIGHT HALF OF THAT CAVITY. In figure it resembled a flattenened oval, or rather the shape of a kidney, prodigiously enlarged, for which, at first, it was mistaken. It assumed a livid or a purple appearance, and was covered anteriorly by the peritonæum. It had displaced about six inches of the colon from

* Frederick Bailey, M. B. Physician to the Reading Dispensary.

its situation in the right lumbar region, which was found lying obliquely across the central part of the abdomen. It adhered very strongly to this gut, at its back part, by means of copious exudations of coagulable lymph, which possessed a remarkably livid appearance; and at that portion of the peritonæum which was contiguous to the tumour, was much thickened by similar exudations. On cutting into the tumour, it was discovered to consist entirely of a BLACK GORE, considerably FIRMER than the crassamentum of the blood, and weighing, as nearly as could be conjectured, about four pounds. At its posterior part, the tumour had no covering similar to that it had anteriorly, but the grume lay in contact with the muscles at the back of the abdomen; and, on tracing its course upwards, it was found to communicate with the first lumbar, and three last dorsal vertebræ, all of which were in a CARIOUS CONDITION. The right transverse processes of the three lowermost were absorbed, and also a portion of their bodies on the same side, presenting to the touch a rough and jagged surface. But the 10th vertebra of the back was in a state of the greatest decay. This had lost all its transverse processes, and only a small portion of its body remained. The intervertebral substances seemed to have sustained little or no injury. The bones however were not the only structure that suffered. The muscles that ran contiguous to these carious vertebræ, WANTED THEIR USUAL CHARACTERISTICS. Such parts of them as were in immediate contact with the caries, were converted into A PAPPY OR STEATOMATOUS MATTER, whilst those a little more remote, were changed into a PALE SUBSTANCE, almost destitute of FIBROUS APPEARANCE, and so SOFT as to be VERY EASILY TORN. The right kidney was pushed a little upwards. The liver looked pale, but the gall bladder was distended with bile. The urinary bladder exhibited strong proofs of inflammation in the peritonæal covering of its fundus, and its inner surface was much loaded with mucus. In the right groin I observed two or three enlarged glands."

10. *Spiculæ of Bone in the Stomach.** A gentleman at dinner swallowed a small fragment of bone. In the evening he felt pain in the centre of the scrobiculus cordis, with sense of anxiety, with periods of remission and aggravation, in the latter his pulse becoming small, hard, and quick, with a cold sweat over the surface, and intense anxiety. Change of posture produced occasional relief. All these symptoms quickly vanished by the use of diluted muriatic acid. Some other cases are related, of similar tendency, and with equal success. The acid should be taken as strong as the stomach will bear it, and pretty frequently, that a solvent action may

* By Joseph M'Sweeny, M. D. *Ed. Journal*, 62.

be kept up upon the foreign substance. These cases do great credit to Dr. M'Sweeny.

11. *Relaxed Rectum.** We are sorry we cannot coincide in sentiment with Mr. Chevalier, "that an inflammatory excess of action in the vessels of a part, is always *accompanied* with a loss of its tone." We can very well conceive that a loss of tone *succeeds* high inflammation in the intestinal coats, as well as in other structures of the body, but we are far from believing that *distention*, from loss of tone, is the *concomitant and effect* of strong peritonæal inflammation. In the latter complaint the function of the mucous membrane of the gut is so much deranged that great quantities of aeriform fluids are rapidly formed, and these, by distending some portions of the canal cause irritation and spasmodic contraction, or twistings in other portions, which lock up the gazeous productions, and greatly tend to increase the distress of the patient, as well as aggravate the peritonæal inflammation.

Mr Chevalier admits that parts of the intestinal canal may become relaxed, and consequently distended, without inflammation, probably from mere loss of tone. The transverse arch of the colon is peculiarly liable to this affection. The extreme distention of the abdomen in tympanites, Mr. C. thinks, is chiefly seated here, and a less degree of it frequently accompanies ascites, occasioning an apprehension, in the mind of the physician, that there is a much larger quantity of fluid in the abdomen than actually exists. This circumstance is to be borne in mind by the surgeon, "who might otherwise be induced to tap the patient, and puncture the intestine by his trocar."

"In such instances, the fluctuation is more obscurely perceived, and it is chiefly at the lower part of the abdomen : the enlargement is greater above the navel than below it; and when the upper part of the belly is struck gently, by the hand, it gives that peculiar sensation and sound which a membranous cavity filled with air communicates."

In many cases of this kind our author has refused to operate, having found, in those bodies which he had an opportunity of examining, after death, that the quantity of serous effusion has been much less than was suspected, and that it was, moreover, irregularly encysted among partial adhesions

* Observations on the relaxed Rectum. By Thomas Chevalier, Esq. F. R. S. &c. &c. *Med. Chir. Trans.*

which had been excited by the irritation of some visceral disease.

> "If it should be thought adviseable in such a case to puncture the abdomen, the operation should be performed by the cautious introduction of a lancet through the linea alba, and not by a trocar."

Dilatations occasionally take place in the sigmoid flexure of the colon, where scybala may be retained for a considerable time by the valvular projections of its internal coat, although a tolerably regular evacuation of fæces may be daily going on.

The prolapsus of a relaxed portion of rectum is often observed and well known; but Mr. Chevalier does not think that practitioners are so well aware that the gut is subject to an excessive dilatation within the pelvis, and to a semi-prolapsus of its upper into the lower part. Such a state of the bowel, however, our author asserts, does frequently occur, producing very distressing symptoms, and especially proving a common cause of that obstinate and habitual costiveness, under which some persons continually labour.

The lower portion of rectum is easily distensible; but, while in its natural state, the peculiar sensibility of its mucous membrane speedily excites it, when moderately distended, to expel its contents; and its muscular fibres are competent to this office (unless the fæces are unnaturally hard) with a very moderate assistance from the abdominal muscles. It is needless to say that it is very important to health, to preserve the sensibility of this portion of intestine unimpaired by strict attention to the regular performance of its functions. If this be long neglected, the natural sensibility gradually diminishes; the bowel becomes surcharged for an undue time, and the energy of the muscular fibres is impaired, so as to require a more forcible exertion of the abdominal muscles to expel the stools; and even this, sometimes, cannot be done without the assistance of medicine.

Under these circumstances, the superior portion of the rectum, and the lower part of the colon become sometimes so overloaded, and, at the same time, so deficient in action, that a great exertion of the abdominal muscles is excited for the propulsion of the fæces, when the upper, and undilated portion of the rectum is forced downwards into the lower and dilated portion, where it may be distinctly felt like a loose bag, of which it is difficult to detect the aperture by finger or bougie. The stools are now voided with difficulty, and in small irregularly shaped pieces, attended often with tenesmus, piles, or an increased secretion from the inner surface of the intestine. In men, the irritation is frequently communicated to the prostate gland, and neck of the bladder. In other instances, especially in females, the parts become so relaxed as to allow of a sufficient accumulation of fæces to fill up the whole pelvis, while the patient is very unconscious of such an accumulation as the following case will shew:—

A lady, who was afflicted with cancer of the breast, was confined to bed by severe pain in the loins; soon after which, she became unable to pass her urine, and it was drawn off at proper intervals by the catheter. Yet, on enquiry, she asserted that her stools were regularly evacuated, and in sufficient quantity. In about a fortnight after this, her attendants one day observed that the anus was dilated to the size of a half-crown, by the protrusion of fæces, which had so stuffed the rectum as to completely choak up the pelvis; and although not hardened, the quantity prevented their being removed without the assistance of instruments. We have met with a similar case ourselves, and we know from dissections which we have made, that hardened fæces will lurk in the cells of the colon, particularly about its sigmoid flexure, for months—we had almost said years, while the more fluid fæces daily pass them and are evacuated per anum. These fæcal deposits nevertheless keep up a constant *organic* irritation in the system which the patient is totally unable to describe, or describes in a way that is more likely to lead the physician astray than direct him to the true source of irritation. This is one among the many inconveniences which would be obviated, were people in the habit of using lavements here, as they are in France.

When an upper portion of rectum is forced down into a lower, as has been described, the lower part of the colon is kept in a state of irritation, and an obscure heavy pain is felt in the loins, and about the sacrum, with such difficulty of voiding the fæces as often leads to the suspicion of stricture. Under these circumstances, an increased secretion of mucus from the surface of the colon may take place in a considerable amount, so as to collect in some of its sacculated portions, and to be discharged in a large quantity, and of a yellowish appearance resembling pus, causing a belief that an abscess had burst internally. The matter, Mr. Chevalier observes, is more tenacious than true pus, is not mixed with blood, and the discharge does not go on, as in case of abscess. If the state of the bowels be now properly attended to, all may do well.

This affection is most incident to females and to those who lead a sedentary life, overlooking irregularities of action in the bowels, and deferring obedience to the calls of Nature. Here purgative medicines are usually had recourse to, and the whole intestinal canal is irritated, for defective action in that very part which is most remote from their influence. The general health now often suffers; all the evils resulting from costiveness taking place, while hypochondriacal gloom and dejection oppress the mind.

In respect to treatment, the principal and most certain relief is to be obtained by the regular use of lavements, till the rectum becomes re-accustomed to empty itself in an *habitual* way.

Where the very lowest part of the rectum continues so dilated as still to allow the upper portion to descend, a strong decoction of oak bark or galls thrown up every night will be attended with the most beneficial effects. If not readily retained, some tincture of opium may be added to it. Should inflammation take place, which sometimes happens, at the prolapsed part, so as to consolidate the surfaces together, a permanent stricture or obstruction is formed, which, by the frequent irritation necessarily attending it, may take on a cancerous character, and of course prove fatal.

This paper, upon the whole, is a very interesting one, and therefore we have given a minute analysis of it.

12. *Entero-Epiplocele.** A gentleman, aged 42, was affected with a double scrotal hernia, one of which was reducible; the other was irreducible, the size of a large fist—the irreducible part consisting of omentum adhering to its sac. For two years it had been troublesome, painful, and, during the late summer heat, had increased in size, apparently from a recent descent of intestine. In a consultation of Messrs. Brodie, Bampfield, and Prower, the patient was recommended to try the horizontal position for two months, to which he consented. Four times a week Mr. Bampfield perseveringly employed the taxis, as long as the patient could bear the pain it occasioned. He was also enjoined to support the omentum, and employ as much pressure with his hands as he could in the proper direction of the ring. A draught of ol. ricini was ordered twice a week. In a fortnight the rupture was more than half-reduced. In three weeks it was pushed entirely into the abdomen. This case is very creditable to the well-known talents of its excellent author.

* R. W. Bampfield. *Med. and Phys. Journ.* 260.

13. *Ovario-Gestation.** Fortunately these melancholy accidents are rare, for Nature is generally correct in all her reproductive operations. The victim to the present aberration was a lady, 39 years of age, who died on the 9th June, 1819, and was examined by Dr. Granville. She had suffered severely, and almost uninterruptedly, from the 12th of the preceding December. The abdomen being laid open, several pints of a fluid resembling blood were discovered filling every space not occupied by the viscera. Many large coagula of blood lay dispersed over the surface, or among the convolutions of the intestines. A tumour, four times the size of a hen's egg, partaking of the general black-reddish hue of the surrounding parts, obscured the view of the pelvic viscera, even after the intestinal mass had been removed.

"A blood-vessel of the size of a large crow-quill, which penetrated the dense portion of the tumour, was traced upwards to the descending aorta, and was ascertained to be a branch of the left spermatic artery. A smaller and much shorter vessel, arising from the tumour, was also found to communicate with the spermatic vein; thus establishing a complete circulation to and from the parts. The inferior and left half portion of the tumour presented a surface consisting, in two or three places, of diaphanous membranes, through which a fœtus of about four months growth was readily discovered." 2.

The left ovarium was the seat of the tumour which progressively distended the coverings of that organ, and at length burst it in several places, when the membranous sac forming the tumour protruded partially into the cavity of the abdomen.

"During this destructive process, that part of the covering of the ovarium was also lacerated, over the inner surface of which the placenta was engrafted, so as to tear the adhesions of the latter, thereby producing that sudden and fatal hæmorrhage which destroyed the life of the mother and the child, and filled the cavity of the abdomen with blood." 3.

The womb had acquired a considerable development during the increase of the fœtus, so as nearly to have reached the size which it usually attains in the fourth month of regular utero-gestation. Its parietes were thickened in proportion—its orifice was closed—but within its cavity neither

* A case of the human fœtus found in the ovarium, of the size it usually acquires at the end of the fourth month. By A. B. Granville, M. D. &c. &c. &c. *Philosophical Transactions.*

fluid, membrane, nor production of any kind was found.* Two beautiful engravings by Bauer illustrate the text of Dr. Granville's paper.

14. *Uterine Hæmorrhage.*† In a case of very lingering first labour, and after the child's head had been impacted in the pelvis for ten hours, with little or no advance, and when the uterine efforts were decreasing rather than increasing, Dr. Campbell applied the forceps, (and we sincerely think in this case justifiably, though we are no great advocates for the forceps in general) and delivered the head after an hour's strong exertion. The uterine efforts were still unequal to the expulsion of the body, and manual assistance was given in its extraction. After exhibiting half a wine glassful of brandy, as a cordial, the hand was placed on the abdomen, and the uterus could be felt "like an immense flattened pouch;" the placenta also could be readily recognized within it. Frictions over the abdomen, and a moderate degree of extracting force applied to the chord, had no influence in exciting the action of the uterus. On pressing the hand on the latter organ, a gurgling noise in the passages was heard, and a stream of blood immediately issued over the bed-side. The appearance of the patient and the state of the pulse corresponded with this alarming phenomenon. The hand was instantly introduced into the uterus, which was pouring out blood in profuse quantities, and, on reaching the placenta, Dr. C. discovered that the greater part of it was detached—thus causing the hæmorrhage. Pressure with the back of the hand on the internal surface of the uterus had no effect in rousing it to action. The Dr. therefore immediately, and with ease, extracted the whole of the placenta. The flow of blood, nevertheless, continued unabated although the clenched hand was pressed, in different directions, on the bleeding surface, and the patient appeared to be sinking. The pulse left the wrist for about twenty minutes, and universal coldness prevailed. The patient lay insensible, but still breathing under great restraint. Another practitioner was now sent for; but before he arrived, the uterus began to contract and restrain the hæmorrhage. The pulse was returning, and the patient began to give faint answers to ques-

* The lady had been the mother of seven children. She had menstruated regularly up to December, when conception took place, from which time there were occasional irregular discharges of a coloured fluid from the vagina.

† Dr. W. Campbell. *Ed. Journal*, 65.

tions. The other practitioner grasped the uterus through the abdominal parietes, while Dr. C. pressed with his hand on the concave surface of the organ. It was full an hour before the hand could be withdrawn, and with it several coagula came away. About 64 ounces of blood were lost.

*From the moment that the hæmorrhage was noticed*, Dr. C. forced the patient to swallow a wine-glassful of undiluted whiskey, *every five minutes*, until the pulse returned—making in all about thirteen ounces of spirits. Injections of spirits and water were also thrown up the rectum. No bad consequences followed, and the lady had a rapid recovery.

Dr. Campbell, as appears to us, did right in proceeding to extract the child by means of the forceps. Whether it was afterwards necessary or proper to give the half glass of brandy, the account is hardly sufficiently explicit to shew. As a general rule, we think it injudicious to give a powerful stimulant immediately after the child is born, but circumstances may undoubtedly render this a very necessary practice.

There seems to have been quite cause enough, for introducing the hand to extract the placenta; this operation is always very much facilitated by making an assistant press with both hands equally upon the abdomen, for hereby the uterus is kept more steady for the operation, and a disposition to contract is given to the uterus, on which the success of the operation mainly depends. It does not appear from the account, that this very necessary part of the process was attended to. We are not aware that "the clenched hand pressed in different directions on the bleeding surface of the uterus," is likely to excite contraction; irritating the *os uteri* seems to be a more effectual mode. The late Mr. Cruickshank used to recommend, that a sponge dipped in vinegar and water should be carried by the hand into the uterus, and the liquor be squeezed out into its cavity, with the view of producing contraction, this we have never tried; but have introduced rags, dipped in vinegar and water, or, what we prefer, port wine, into the vagina for the same purpose, and often very successfully.

A wine-glassful of undiluted whiskey is forced upon the patient every five minutes, "from the moment the hæmorrhage was noticed." Had this been given from the moment syncope occurred, we should not have objected to it. But to employ cordials and stimulants during the active state of hæmorrhage, not only seems to us uncalled for, but positively hurtful. The generally adopted practice of cooling the apartment and the patient; of applying cold vinegar and water over the abdomen and pubis; of making pressure upon the uterus; of introducing ice into the vagina, or, as before mentioned, port wine, seems to us much more proper practice. The exposure to cold, however, we have repeatedly seen carried too far; it has been persisted in, after the hæmorrhage has ceased, or become very passive, and syncope has come on. At such a time the cordializing plan requires to be adopted.

We are not very strong advocates for opium, either solid or fluid, in uterine hæmorrhages. But as a remedy for some of the consequences of hæmorrhage, nay, perhaps for some symptoms attendant on the state of hæmorrhage, it may be occasionally useful. The restlessness and irritability consequent to, or accompanying, uterine hæmorrhage, particularly, is perhaps best quieted by laudanum.

If we are right in our opinion, respecting the use of laudanum in floodings, there must be much mischief occasioned by its so general and promiscuous use. But names of such high authority are mentioned in favor of it, as almost leads us to doubt the accuracy of our own observations and reasoning.

15. *Hernia.**—Case. "Mrs. Seruya, ætat 60, of emaciated form, and infirm constitution, had been afflicted with ascites, and femoral hernia on the left side, for some time. On the 13th September, 1818, she experienced a sudden descent of the rupture, accompanied with violent pain, syncope, and vomiting. The usual symptoms of strangulated hernia ensued, and after various trials by the taxis, it was agreed, in consultation with Messrs. Barker and Donnet, of Gibraltar, to operate. The operation was accordingly performed at seven in the evening, by candle light. On laying open the sac, the stricture at Poupart's ligament was found very firm. '*I dilated it obliquely upwards and inwards*, and reduced the gut. Common dressings were applied. At ten o'clock we were sent for, as an alarming hæmorrhage had taken place. We found the patient much exhausted—covered with cold clammy sweats—pulse scarcely perceptible. In short, she had every appearance of approaching dissolution. The bedding was completely soaked with blood. The dressings were removed, and sponge, with graduated compresses, supported by a firm bandage, applied. The hæmorrhage continued, and we had no alternative (the reduced state of the patient not admitting time to search for the artery) but to trust to compression by the hands, which was continued during the whole night, and part of next day.' Every time the hand was withdrawn, the blood

* Case of violent hæmorrhage, after an operation for strangulated hernia, where long-continued pressure succeeded. By William Mathias, Surgeon, Gibraltar.

flowed. At the end of 48 hours, the compresses were removed, and no hæmorrhage returned. But she remained in a very doubtful state for some time, being subject to palpitations of the heart and syncope. Ultimately, however, she entirely recovered."

What artery was cut in this instance? Is it a good direction to *cut upwards and inwards?* We believe the direction should be *directly upwards*, both in femoral and inguinal hernia.—*Rev.*

16. *Wounds of the Intestines.** Baron Larrey has had extensive opportunities of observing these sorts of lesion from musket shots, and their susceptibility of cure, by the resources of nature, assisted by art. Suppose a projectile body penetrates the abdomen of an adult, and destroys a portion of ileum or colon in its track. The contusion of the parts struck is necessarily accompanied by some tumefaction, and a degree of contraction in the trajet of the ball. The intestinal contents either find their way through the external wound, or lodge in the track of the wounding body, without communicating with the general cavity of the abdomen. To assist Nature in her work, the Baron is careful in searching for the wounded part of the intestine, in order to bring it to the edge of the external opening, and confine it there by a loop of thread passed through the mesentery.

"We have had occasion to attend after many battles to a great number of soldiers wounded in this way, the cure of all of which have generally been conducted in the following manner:—Up to the period of the separation of the eschars, or sloughs, the alvine evacuations pass in small quantities by the wound; but afterwards, finding no obstacle, they issue freely, and continue till the entire cleansing of the wound; at that period, it becomes proper to favour the approach of the edges of the two wounds, both those of the external parietes of the abdomen, and those of the wound in the intes-

* Observation sur une plaie du bas ventre, avec lesion d'un intestin grêle: précédée de quelques reflexions sur les plaies des intestins en general. Par M. LE BARON LARREY. *Journal of Foreign Medicine and Surgery*, *No* VIII. *for October*, 1820, *and* LA REVUE MEDICALE, *for July* 1820.

☞ We may here take an opportunity of expressing our joy at seeing our respected cotemporary, the *Quarterly Journal of Foreign Medicine*, again return, after two months absence, *without leave*. Among many other interesting articles in the last number, we beg to draw the attention of our brethren to an able paper on Climate, and another on Yellow Fever. We confidently hope that the Profession generally, will patronize this useful and meritorious Journal, which evinces equal honour and ability in its conductors.

tine, by aid of slips of sticking plaster (Bandelettes agglutinatives), and a slightly compressive concentric bandage—the two wounds approach each other gradually and at the same time—the parts homogeneous to each other naturally adapt themselves, contract a mutual adhesion, and form the cicatrix, which first displays itself on the wound of the intestine, and so gradually extends itself from within to the exterior—the intestine itself also experiences a degree of contraction correlative to the original loss of substance.

"The greater part of the early adhesions disappear or become effaced in succession, and those parts which had experienced any transposition recover their natural and respective position, with the exercise of their functions; phænomena which we have already taken notice of in wounds of the belly attended with protrusion of the epiploon." *Journal of Foreign Med. p.* 391.

When the intestines are wounded by a sharp or polished weapon, they do not present the same phenomena. They are, our author thinks, more dangerous, and require prompter assistance, than in cases of wounds from balls.

"Two main proceedings are pointed out in their cure: the one consists in retaining the wounded portion of the intestine, at the lower part of the wound of the belly, by means of a loop (anse) passed into the mesentery, in order to prevent any effusion of the alvine matters into the abdominal cavity; and to give time for nature to isolate the wounded intestine, (in like manner as happens in wounds from fire arms) till the causes of irritation are entirely subsided; till the lesed parts gradually recover their original position, and the lips of the intestinal wound mutually approach so as to become cicatrized, for this is the true cure effected or attempted by nature: and this, without doubt, is the most advantageous and the most ready mode of proceeding." 391.

The second indication consists in a kind of invagination of the wounded intestine, by means of a simple suture, "aided and supported by loops of thread, passed into the substance of the two ends of the wound of the intestine, and sometimes by interior ones."

Our author admonishes us to be careful to comprehend the least possible quantity of the intestinal tube within the points of the suture, lest we diminish the calibre of the bowel, and thus obstruct the passage of the alvine contents.

The suture is to be made by the over-cast-stitch (à point par-dessus) and the threads ought to be waxed and smeared with some mild cerate. They ought also to be of sufficient length to come outside of the wound, there to be retained till the time of their extraction, which is from the fifth till the seventh or ninth day. It is necessary to state, that the Baron uses a *double* glover's stitch, one thread being black and the other white, a complication, in our opinion, equally troublesome and unnecessary. The Baron directs us to replace the knuckle of intestine within the cavity of the abdomen, "in such a manner that it may move itself freely; because if we retain it on the edge of the exterior wound, we shall cause it to suffer a series of flexures, which will impede the course of the fæces."

The slight adhesions which take place between the edges of the wound and the ambient parts are, the Baron asserts, only temporary, and are insensibly separated by Nature, in the progress of the cure.

Inflammation, of course, constantly supervenes on the mechanical irritation produced by the suture, and to combat this, the Baron affirms that nothing more is necessary, than cupping glasses with scarifications, promptly applied "in a parrallel series from the upper to the lower part of the belly, *imitating the march of the galvanic fluid from the positive to the negative pole;* (a curious idea truly) and these applications must be repeated as often as may seem necessary."

"We ought to follow up these local depletives and derivatives, with oily embrocations, and tepid bathings, emollient glysters, iced and mucilaginous drinks taken in small quantities and often repeated —general bloodletting is rarely necessary."

General blood-letting may not be necessary in France, but we should not like to trust to local detractions of blood in this country, when the abdomen was penetrated and an intestine wounded.* Still farther are we from agreeing with Baron Larry, that cupping the abdomen is the sole treatment required in the yellow fever. The Baron knows little of this disease, or he would not propose any such measure, excepting as an auxiliary.

We shall considerably abridge the following case, which the Baron introduces in illustration of the foregoing precepts.

Jean Jolin, 25 years of age, fell on his own sword (a naked sabre) on the 27th April, 1820, and received a deeep wound in the abdomen. Being taken to the neighbouring village of Picteau, Dr. Carré found the wound fifteen lines in length, running in a transverse direction on the lower part and right side of the belly, attended with a strong protrusion of the ileum, which had already become tumefied. The man had nausea and vomiting. On examining the protruded intestine, M. Carré found a wound in it of some extent discharging stercoraceous matter. He closed it by the glover's suture, and replaced it in the ventral cavity. The wounded man was

* See the second edition of Dr. Hennen's excellent work on Military Surgery, *page* 401 *et seq.*

then dressed, and, strange to say, he was sent off to the Military Hospital in Paris. "During his journey, which was a very painful one, he had many copious vomitings and a bloody alvine evacuation." We never heard of such imprudent (we had almost said inhuman) conduct, as the sending a man with wounded intestine, to a distance, where no military movement required such a step. On arrival at Paris, the surgeon of the guard discovered a tumefied portion of the small intestine protruded, without any appearance of wound, which he immediately returned. Dressings were applied, and emollient drinks and glyters were exhibited. The patient was greatly debilitated, and spent the night in permanent anxiety, with bilious vomitings, violent colicky pains, tenesmus, and slight sanguineous discharge per anum.

Next morning the Baron dilated the wound, which enabled him "to discover through a considerable sanguineous accumulation established behind the wound and in the peritonæal cavity, many circumvolutions of the intestine, which had already contracted adhesions one with the other." Although there were strong symptoms of internal strangulation, they durst not tear these adhesions to get at the strangulated portion of bowel. They contented themselves with evacuating the blood effused into this kind of reservoir, and dressing the wound with linen, in which holes were cut.

Many very bad symptoms now came on, as small and frequent pulse, pale countenance, dull watery eye, coldness of the extremities, frequent nausea and vomiting, evacuations tinged with blood, abdominal pain and tympanitic distension. Cupping was now employed, and by the time that the third or fourth glass was applied, the tympanitic state of the belly sensibly diminished. The patient experienced relief, and, in a little time, passed by stool several evacuations of a biliary and sanguineous character. The cupping was repeated all over the abdomen, and then camphorated oily embrocations, followed by light anodyne cataplasms, glysters, &c.

The night was tolerable; but in the morning several bad symptoms returned, especially the colic pains and vomitings. Again a sensible improvement was obtained by cupping, which lasted eight or nine hours, when the bad symptoms returned with such violence that they did not expect the patient to live from one moment to another. The cupping glasses, with and without scarificators, were repeatedly applied, which, with the aid of sedatives and mild purgatives, produced a visible amelioration. A blister was applied all over the abdomen. They had now arrived at the eleventh day, on the night of which "the disease came to a crisis by two phlegmons, which formed in the regions of the parotids. From that moment all the inflammatory symptoms of the belly almost in an instant disappeared, and the patient, on the 13th day, had copious evacuations of fœcal matter." A thread, three inches and a half long, now issued from the wound of the belly. He was completely cured by the 60th day.

We believe that English surgery would not have interfered so much with the efforts of Nature in this case, and that English surgeons are, upon the whole, more cautious of the scalpel and needle in cases of wounded intestines. We wish, however, to draw the attention of our brethren here to the extent to which cupping was carried, and the unequivocally good effects of this measure. We are confident that *local* bleeding is still too much neglected in this country, and on that account we are anxious to bring forward those cases which illustrate its beneficial consequences. In the class of injuries here descanted on, however, we are far from wishing to recommend an *exclusive* reliance on *topical* blood-letting. We have witnessed too often the powerful control which venesection exerts over abdominal inflammation, to discard such a measure from our methodus medendi.

17. *Bursting of the Urethra**—This is a most serious accident, and, as Mr. Bell justly observes, belongs to the higher departments of surgery, requiring a perfect acquaintance with the principles of the art, and a dexterous hand—one not partial to operations, yet not hesitating to do what appears bold, when the occasion calls for it.

" When the patient has had a stricture attended with much irritation in the perineum and neck of the bladder—when he has to strain and force to pass a few drops of urine—when the urine feels scalding hot—when the patient, on closing his legs, has a sensation of a tumour betwixt his thighs, though there be no such tumour there; then he is in danger of an extravasation of urine." 198.

If a patient with an irritable stricture has had the bougie introduced in such a manner as to induce inflammation and strangury, followed by cold shiverings, and then a hot stage —when there is a sensation of tenderness, heat, and swelling in the perineum, with violent forcing pains to make water, there is danger of the inner membrane of the urethra giving

* Mr. Charles Bell's Treatise on the Diseases of the Urethra, Vesica Urinaria, Prostate, and Rectum. New Edition, 1820.

way and permitting the urine to escape into the cellular texture of the scrotum and penis.

After these symptoms have continued some time, the patient feels that, at last, the water is flowing; but it does not appear outwardly. By and bye, the scrotum becomes enormously distended, the patient is seized with shivering, &c. If the swelling be not immediately relieved by incisions, the integuments of the penis are distended, and the extravasated urine will spread over the pubes, lower part of the abdomen, and even the loins. The consequences are often terrible. A dark inflammation affects the skin that is undermined with urine —the skin sloughs—the whole scrotum separates, and leaves the testicle exposed.

Mean time the violence of the fever subsides—the pulse is quick and feeble—the countenance changed—the features shrunk—and gastric irritability with hiccup sets in. The patient sinks, if not supported. Mr. Bell, after relating a great number of most interesting cases, which cannot be too often studied by the surgeon, makes the following observations:—

"1. It appears that punctures of the scrotum are insufficient even to empty the cellular texture of the extravasated urine, and quite unfit for preventing the urine taking the same course a second time. If the lancet be used, the shoulder must be moved, while the point is kept at rest, so as to make a large opening in the skin.

"2. For the most part, the urine bursts into the perineum, and is carried by the fascia of the perineum forward into the looser scrotum. In this case the opening into the scrotum must be at the back part, and the point of the instrument directed backwards, so as to cut freely through the fascia, and give issue to the urine as it escapes from the perineum.

"3. But it will be seen here, that the extravasation takes place sometimes more anteriorly, and the *œdema* of the preputium is the first sign of the approaching danger. In all cases, therefore, it is proper to sound the urethra with a bougie (and this should be done in the gentlest manner,) to ascertain the place of stricture, that the puncture may be directed with reference to the spot from whence the urine issues from the urethra, and which is always behind the stricture.

"4. The urine has a deadening effect on the cellular membrane, when it is permitted to fill the integuments. When in a smaller quantity, and with diminished force, it produces a blush of erysipelas, which subsides and rises again in the form of more phlegmonous inflammation. This was particularly the case in two instances, and the fever and the hard swelling of the skin required cold and sedative applications.

"5. In most of these cases, the yielding of the urethra was preceded by a state of much excitement and irritation. An ulceration of the urethra is a consequence of this irritation, and the membrane is thereby weakened. The push of urine bursts through this tender part, before there is consolidation of the surrounding parts, or before the cells of the common texture are glued together by the process of inflammation. Hence there is no limit to the flow of urine, and hence the dangerous nature of the accident: for the general powers of the system quickly sympathize with the death of the part, and fall low; and there is a just apprehension of the patient sinking.

"6. The circumstance of irritation preceding the rupture, teaches us to be particularly cautious either of exciting the urethra by interference with instruments, or of permitting a fever to be raised by imprudence on the part of the patient, in a certain state of stricture with irritation. I need not here repeat what may be the dreadful consequences." 237.

This view of the subject teaches us also that when the urethra is burst, and free passage is given to the urine, we must make it a principal object to allay irritation. It sometimes happens too, that after we have made a free passage for the urine, the integuments of the penis are again puffed up by a *serous* effusion, the consequence of inflammation.

If we see this state early, or when forming, it is best treated by cold applications—if late, or when formed, with tepid fomentations. If it be confounded with a urinous tumefaction, and a catheter introduced to draw off the water, the swelling will increase, and terminate in suppuration.

Upon the whole, this section of Mr. Bell's work abounds in important matter and solid instruction for the practical surgeon.

18. *Dysmenorrhœa.** Dr. Dewees considers it to be a matter justly challenging surprize, that this painful affection of the uterus should have attracted so little notice from the earlier writers on medicine, while almost the whole of the more recent authors are involved in the same charge of supineness towards a disease deserving serious attention. The relief of pain is but a subordinate consideration. The complaint removed, the married woman, hitherto doomed to barrenness, becomes fruitful, and fulfils the end of her creation. It is to Dr. Denman we are chiefly indebted for the history and real character of this complaint. Drs. Cullen and Fothergill have mentioned the affection; but neither of them has adverted to its most remarkable attendant—the discharge of a membrane.

* On Dysmenorrhœa. By Wm. P. Dewees, M.D. *American Medical Recorder, Vol.* I.

Morgagni,* long before Dr. Denman, had given a very curious case of this kind, which was overlooked by Dr. D. The latter, although he notices the formation and escape of this deciduous substance, attempts no explanation of its origin or the consequences to which it gives rise. He merely states that "the pain is to be attributed to an increased degree of irritability in the habit, or to the difficulty with which those vessels designed for the menstruous discharge became permeable," which is saying little. Dr. Fothergill's explanation runs thus:—

"This excruciating pain seems to be spasmodic, and to proceed from the extreme irritability of the uterine system: the blood naturally determined hither, in order to its being discharged, by distending the very irritable vessels, occasions the spasms; this produces a constriction of the vessels; they become impervious, and the nisus to the discharge continuing, the pain becomes exquisite and general, till the patient, worn out with the struggle, is debilitated and sunk; the fluids are then dismissed, some ease succeeds, but the patient is often so reduced as not to recover her usual strength before she has another conflict to undergo."† *Medical Recorder*, *p.* 156.

Dr. Fothergill and some other physicians have remarked that sterility generally accompanies the complaint. The following extract contains Dr. Dewees' description of the affection under consideration.

"This complaint for the most part commences in women who are obnoxious to it, with the first menstrual periods, and unless prevented, most pertinaciously continues at every subsequent return of the Catamenia.‡ We have never observed any particular constitution or temperament especially liable to it. We have witnessed it both in the delicate and robust; in the sanguineous and the phlegmatic. The discharge commences sparingly for some time, and is then for a short period, almost altogether arrested; so soon as this happens, pain is felt, and this returns and intermits like the pains of labour—after a continuance of these alternate pains for an uncertain period, relief is sometimes suddenly experienced, and there is found discharged from the vagina a membraneous substance, of uncertain size,—sometimes it resembles when spread out the form of the uterus; at other times it is broken into fragments, but always maintains its membraneous texture.§ So soon as this membrane is com-

---

* Epist. xcviii. Art. 12.

† Fothergill's Works, p. 468.

‡ The woman may however become subject to this complaint at any period almost of the menstruating time of life. I have, in more instances than one, known it to follow abortion.

§ Dr. Denman declares this membrane to be smooth on one side and pletely thrown off, the woman is relieved, unless there be a fresh production of this substance to stimulate the uterus to new exertions, and to new torments; this is by no means unusual, and several days are sometimes employed before these efforts cease—at other times a few hours are all that is required to restore the woman to tranquillity. It is remarkable, that the quantum of pain is not always in proportion to the quantity of membrane discharged—we have seen extreme torture from a very small portion, and less pain where the deciduous product was considerable. But this is not difficult to account for.

The virgin and the married woman are equally the victims of this distressing complaint. We have known it to commence immediately after marriage where it had not previously existed; and on the other hand, we knew it once to cease after this consummation.

Beside the alternate pains which we have just noticed, there is almost always a distressing aching in the back and hips, and which almost invariably announces the approach of the period; nor does this cease, in many instances, until two or three days have elapsed after the catamenial flow." 158.

In attempting to account for the formation of the deciduous membrane above described, Dr. Dewees observes that it is now generally agreed that the menstrual discharge is not blood, but a peculiar secretion. Among other differences between it and blood, may be mentioned, 1st. that it is thicker. 2d. that it does not resemble blood in smell. 3d. that is much darker coloured. 4th. that it never separates into its constituent parts. 5th. that it never coagulates. 6th. that it is not near so susceptible of the putrefactive process.

Our author considers it as a most wise provision of Nature that the uterine vessels, in a healthy state, operate such a change on the lymph of the blood, that it is incapable of coagulation in the womb, otherwise the most serious inconvenience would ensue.

" Having (we trust) rendered it more than probable, that the fluid thrown out at the menstrual period is the product of a secretory process; and that this process is instituted with the view to deprive the coagulating lymph of the power of coagulation; and that when this secretion is healthily performed this end is uniformly affected; let us advert to the consequences that would follow, supposing that, from some cause or other, an interruption is given to this healthy condition of the uterus: it would seem, under such circumstances, to follow as a consequence, that the fluid discharged would differ from the product of a healthy and well established secretion. The process would be imperfectly performed, and the required changes would not be completely induced, the coagulat-

---

floculent on the other; and this observation is confirmed by my friend Dr. Horner, who kindly examined a portion of it for me.

ing lymph would not be entirely deprived of its usual or common capacity, consequently the menstruous fluid would be imperfectly elaborated; so soon then as this fluid is eliminated from the secretory vessels it will begin to separate into its constituent parts, the colouring matter will separate from the imperfectly subdued coagulating lymph, and will, from its superior density occupy the lower or most depending part of the uterine cavity, and will, sooner or later, make its escape, while the coagulating lymph will remain either altogether or in part to spread itself over the internal face of the womb, and will, as it is wont to do when in contact with living parts, quickly assume the appearance and density of membrane.

This membrane will be, to all intents and purposes, an extraneous substance to the uterus, and will consequently stimulate it to the effort of throwing it off, which will be eventually effected by the institution of alternate contractions; and *hence the pain* during this process." 161.

This reasoning is ingenious; but what is more interesting is the fact that Dr. Dewees has found tinctura guaiaci an effectual medicine in stimulating the uterus to throw off the deciduous membrane, and prevent its subsequent formation.

19. *Fractured Femur.** The unexpected success, and the equally unexpected disappointments, which Dr. Colles met with, in the treatment of fractures of the neck of the femur, induced him, a few years ago, to turn his attention more particularly to this injury. The discrepancy abovementioned he had endeavoured to account for by dissection, and the result is herewith communicated to the surgical profession, rather as a stimulus to further investigation, than as a basis for any settled rule of practice.

*Case* 1. Was a *subject* brought to the Anatomical Theatre for dissection, and the history, of course, unknown. The capsular ligament of the hip joint was thicker and closer in texture than natural. The fracture of the neck was near the trochanter, but still within the capsular ligament.

"The fractured surface of the upper piece exhibited many spots apparently covered with a cartilaginous incrustation, which spots, on more close examination, were found owing to the conversion of small portions of the bone into a substance resembling ivory. The lower fractured surface, widely expanded, was formed into a sort of cup, as if the bone had been rendered soft, and, while in that state,

* Fracture of the Neck of the Femur, illustrated by Dissections. By A. Colles, M. D. *Dublin Hospital Reports, Vol.* II.

had been acted upon by the upper piece, which was pressed on it by the weight of the body. One part of the edge of this cup-like surface was formed of two pretty large fragments of bone, which were closely connected to it by a strong ligamentous substance." 336.

The round head of the bone was retained in the acetabulum by the ligamentum teres, and was entire. No intermediate substance held the fractured surfaces in apposition, each being connected with the capsular ligament by very strong ligamentous bands, which passed from the internal surface of the capsule to almost every point of the outer surface of the fractured pieces.

From these appearances, it is obvious that no ossific union had been attempted by nature between the fractured bones, and that the stability of the limb had depended upon the strength of those ligamentous bands by which each piece was connected with the capsular ligament, aided, no doubt, by the extraordinary thickness which the capsule had acquired.

*Case* II. The following appearances were observed in the left thigh of a female corpse. Left femur fractured transversely, on a level with the brim of the acetabulum.

" Two strong ligamentous bands, one arising from the edge of the acetabulum, and the other from the internal surface of the capsular ligament, stretched across to the broken surface of the head of the bone, and seemed as if they had assisted the round ligament in confining the head in the socket. The head of the bone was perfectly sound, as was the ligamentum teres. The two surfaces of the fracture anteriorly admitted of a separation from each other to the extent of an inch, having, at this part, no other connexion than two or three tendinous bands, nearly an inch long, and very distant from each other. Posteriorly these surfaces were united together by a very strong ligamentous substance, which was so connected with the capsula that it appeared as if it were formed by the ligament sending a thick production across between the fractured surfaces. The neck of the femur was evidently shortened." 338.

Eleven cases are related by Dr. Colles, all interesting in themselves, but too long for analysis here. We shall subjoin a few remarks with which our author closes the paper.

" It is very plain that a fracture may take place either near the edge of the acetabulum, or in any part of the length of the neck of the femur, and also that a fracture may take place close to the capsular ligament, and yet exterior to it. The efforts of Nature to repair the injury are independent of the seat of the fracture, and yet they present varieties which have hitherto been overlooked. Thus, in the two first of the foregoing instances, the broken surfaces moved on each other, and were converted into a state approaching to ivory.

No attempt had been made to unite the fracture, and the pieces of bone were held in apposition only by new ligamentous productions from the capsular ligament, which are inserted into the external surfaces of each piece." 352.

The circumstances which Dr. C. found common to all these fractures were, that the capsular ligament was not lacerated—and that, in every instance, the capsule was increased in thickness. Another circumstance attending these fractures, was the removal of all, or the greater part of the neck of the femur, a fact which has been long since observed, but not yet fully explained.

20. *Luxation of the Thigh, in the superior anterior direction.** This rare species of luxation occurred from the fall of a horse on his rider, and is related by Baron Larrey. The attendant phenomena were, limb separated to nearly a right angle from its fellow, and turned in on the pelvis—foot and knee turned outwards, a deep hollow occupying the place of the trochanteric projection—head of the femur projecting under, and stretching the crural vessels of the inguen—the whole limb swelled, spotted, and immoveable, with sense of numbness, coldness, and excruciating pain in the groin and belly. After several ineffectual efforts at reduction, Baron Larrey reduced it himself by suddenly raising with his shoulder the lower extremity of the femur, while with both hands he depressed the head of the bone, which rested before the horizontal portion of the pubis. Inflammation of the joint, with retention of urine, pain in the groin and limb, fever, &c. followed, but were reduced by local bleeding, cataplasms, and the employment of the catheter. The limb remained somewhat longer than the other, when the patient was horizontal, not when erect.

21. *Tabes Mesenterica.*† The symptomatology and pathology of this disease are pretty well known. Chance suggested to Dr. Fletcher a mode of treatment which, in his hands, has proved very successful. It is simply this: after removing the causes, as much as possible, on which the disease depends, a dose of some cathartic medicine is given

---

* Bulletins of the Fac. of Med. No. 1.

† On Tabes Mesenterica. By Dr. J. Fletcher. *American Medical Recorder, Vol.* I.

every day, or every second day, making use at the same time of a tepid bath, twice a day, for 15 minutes each time, composed of a strong decoction of green black oak bark (quercus tinctoria) and clothing the patient well in flannels, if the season require. In pursuing this plan eight or ten days, he always found an evident amendment, and in six or eight weeks, a perfect cure. Several cases are detailed in corroboration, where the tumid abdomens were quickly reduced by this treatment, and the general health restored.

## Figures and Tables

**Fig.1. Fig.2. Fig.3. f1:**